# Addressing Mental Health in a Changing Climate: Incorporating Mental Health Indicators into Climate Change and Health Vulnerability and Adaptation Assessments

**DOI:** 10.3390/ijerph15091806

**Published:** 2018-08-22

**Authors:** Katie Hayes, Blake Poland

**Affiliations:** Dalla Lana School of Public Health, University of Toronto, Toronto, ON M5T 3M7, Canada; blake.poland@utoronto.ca

**Keywords:** climate change, mental health, vulnerability, assessment

## Abstract

A growing number of health authorities around the world are conducting climate change and health vulnerability and adaptation assessments; however, few explore impacts and adaptations related to mental health. We argue for an expanded conceptualization of health that includes both the physiological and psychological aspects of climate change and health. Through a review of the global literature on mental health and climate change, this analytical review explores how mental health can be integrated into climate change and health vulnerability assessments and concludes with recommendations for integrating mental health within climate change and health vulnerability and adaptation assessments.

## 1. Introduction

It is well understood that climate change affects health; most articles addressing the topic area cite the proclamation made in the 2009 Lancet article, Managing the health effects of climate change, that “climate change is the biggest global health threat of the 21st century” [1], where health is understood to mean physical health. The lesser known, and often overlooked, health impact of climate change is the mental health implications from our changing climate. Often, this oversight is due to the general lack of attention to mental health within broader conceptualizations of health, and also to the challenges of monitoring, assessing, and predicting the mental health implications from climate change-related hazards. We argue for an expanded conceptualization of health that includes both the physiological and psychological aspects of climate change and health.

There is also an overarching misunderstanding that mental health equates only to mental illness and mental problems. However, mental health, like physical health, includes states of affirmative health, wellbeing, and emotional resilience, as well as illness. Moreover, mental health is encapsulated in the conceptualization of psychosocial health—the interplay of psychological and social wellbeing [2]. In other words, things that affect our social world like climate change-related income insecurity, food and water insecurity, and conflict and displacement have implications for psychological as well as social wellness. A further consideration is that a holistic understanding of mental health, often found within indigenous ways of knowing, includes spiritual wellbeing and connectedness to nature and one’s environment [3].

Arguably, because the mental health implications from a changing climate are often overlooked, there is a weakened understanding of the burden of illness associated with climate change. A focus on climate change and mental health can help enhance the understanding of factors that may enhance psychosocial resilience. Briefly, psychosocial resilience is defined here following Kumar (2016), as: “the ability of an individual or a system to recover from a setback, adapt well in the face of trauma, and survive and thrive despite significant adversity and stress” [4]. One way to evaluate climate change implications for health is through climate change and health vulnerability and adaptation assessments (CCHVAA). CCHVAAs are policy-relevant tools that capture evidence of the health consequences related to climate change, as well as interventions that support adaptation to a changing climate. While a growing number of health authorities around the world are conducting CCHVAAs, to our knowledge, few explore impacts and adaptations related to mental health. Typically, assessment tools overlook mechanisms that can support assessment-users to capture evidence of the mental health implications of climate change.

Following a brief overview of research on the mental health consequences of climate change, we explore tools and approaches that support assessment-users to monitor, measure, and evaluate the mental health implications of climate change—including mental wellbeing, resilience, mental illness and mental problems. We explore how mental health can be integrated into climate change and health vulnerability assessments, and we conclude with recommendations to support this integration.

## 2. Background

### 2.1. Climate Change and Health

The Lancet’s Commission on Health and Climate Change warns that climate change is affecting health now and these health effects will continue to grow and magnify if efforts to mitigate and adapt to these changes are not addressed globally in a collective and timely manner [5]. Further, these climate change health effects amplify existing inequities—placing marginalized people, who generally have contributed the least to the climate change problem, at the greatest risk [5,6].

While the physiological health impacts of climate are well known, psychological aspects remain relatively unexplored. Some well-known health implications of climate change include: a rise in vector-borne diseases; heat-related morbidity and mortality; injury and illness from extreme weather events; increased cardiovascular disease and increased aeroallergens from poor air quality; and water and food security concerns from water and food-borne illnesses and malnutrition [2,7,8,9]. Lesser known climate change impacts on mental health include direct climate impacts, like extreme heat or extreme weather events (including floods, wildfires, hurricanes, heat waves, etc.); indirect, like the social strain and resource loss related with drought, sea-level rise, melting permafrost, or overarching impacts associated with the knowledge and awareness of climate change impacts on planetary and public health [10].

Notable direct climate-related hazards that affect mental health include: extreme heat, extreme weather events, and morbidity associated with vector-born disease (VBD). Extreme heat has been shown to increase mood and behavioral disorders amongst people with pre-existing mental illness and the elderly who have poor thermoregulation [11,12,13]. Extreme weather events, like flooding, hurricanes, and wildfires have been linked to depression, anxiety, post-traumatic stress disorder (PTSD), suicidal ideation, substance abuse, vicarious trauma, loss of identity and a loss of a sense of place, relationship strain, and helplessness [11,14,15,16,17]. Vector-borne diseases like West Nile Virus and Lyme disease may compound mental health issues for people with pre-existing mental health illness by contributing to cognitive, neurological, and mental health problems [11].

At the community level, climate change-related drought and sea-level rise can threaten natural resources, placing strain on communities, resulting in displacement, violence, and crime [14,15,16,17,18,19]. Further, a loss of land-based activities and occupations, due to a changing climate, can affect personal and community socioeconomic status leading to social and economic instability, loss of community, and a disrupted sense of belonging [14,19]. This is particularly evidenced in the north where climate changes are more pronounced, and their impacts felt more keenly by those living on the land [20].

The overarching awareness of climate change as a threat to well-being and survival is also a source of distress, anxiety, and fear; this awareness, however, may also trigger climate change mitigation and adaptation behaviors that support community and build psychosocial resilience [10,21]. Importantly, there is research that suggests that extreme weather events may also, in some cases, incite compassion, altruism, and trigger post-traumatic growth—wherein trauma may be met with personal strength, a sense of belonging, gratitude for existence, hope, and transformation [22]. The psychosocial outcomes related to a changing climate are thus broad and wide-ranging.

### 2.2. Study Populations

While the mental health implications of climate change can affect anyone, the impacts tend to be amplified in populations who are most marginalized. Much of the current, empirical literature is focused on the mental health implications of climate change for indigenous peoples, compounding existing social inequities stemming from a legacy of colonization. In Australia, researchers have found that indigenous groups tend to be most exposed to the extensive and reoccurring effects of wildfires and drought, which threaten agriculture, food and water supplies, and social and economic wellbeing, leading to a whole host of psychosocial trauma [23,24,25,26,27]. In Canada’s circumpolar north, a team of researchers conducted a multi-year study on the climate change-related mental health implications to Inuit people [20,28]. They found that warming temperatures and melting sea ice affect food security, culture, travel, and autonomy of Inuit people, thus impacting a sense of place and identity and contributing to mental health problems and addictions [20,28]. Other populations disproportionally at risk for the mental health consequences of climate change include: children, seniors, women (particularly pregnant women), resource-based workers (including farmers and fishers), people with low socioeconomic status, and people with related, pre-existing conditions [10,11,14,21]. What is clear is that climate change is an environmental determinant of health that disproportionately affects those who are most marginalized based on existing social determinants of health [5].

### 2.3. Climate Change and Health Vulnerability and Adaptation Assessments

Increasingly, CCHVAAs are being conducted around the world to help inform public health decision-makers about the risks, impacts, and vulnerabilities associated with climate change and health. These assessments also help decision-makers identify opportunities to build or enhance health adaptations to a changing climate, including: public education regarding heat health and vector-borne disease risks and how people can protect themselves from these; and health sector preparation for surge capacities during and after extreme weather events [2]. CCHVAAs can be conducted at all levels from local to federal. In Canada, national climate change and health assessments have been conducted as early as 1997 [29] and Health Canada is preparing for the upcoming release of a national climate change and health assessment in 2021 [30]. As of 2017, nine sub-national (including regional, provincial, and municipal) climate change and health assessments have been completed or are nearing completion in Canada [31]. The US and the UK have federal mandates to prepare CCHVAAs every four to five years so that decision-makers are abreast of the latest science and research [32,33].

The current body of assessment reports present compelling information about the region-specific physical health implications of climate change; however, few assessments present information on mental health effects. Notably, the forthcoming Health Canada assessment is developing a chapter on the mental health consequences of climate change [30]. Another noteworthy exception is the 2016 US Climate Change and Health Assessment that devoted an entire chapter to reviewing the current literature on the topic [11]. In that review, authors point out that there are currently no psychosocial impact assessments that empirically measure and “identify important changes in mental health and well-being associated with climate change” [11] (p. 288). They suggest that psychosocial impact assessments and monitoring programs ought to be implemented within assessments to provide standardized methods to measure and predict the psychosocial outcomes from a changing climate [11]. Fritze et al. (2008) echo these sentiments and voice a concern: “What kinds of indicators, data sets and data analytic techniques will be needed to predict and monitor the health and mental impacts of climate change?” [10].

The discussion section below explores these key questions while noting the challenges and opportunities for incorporating mental health indicators into assessments. We provide guidance on how to incorporate climate change and mental health monitoring and surveillance strategies into CCHVAAs with the aim of supporting a comprehensive valuation of all of the health implications from climate change, as well as to facilitate an understanding of what is needed to support psychosocial resilience and climate change and health adaptation.

## 3. Methods

A global scoping review was conducted on literature at the intersection of climate change, mental health, and psychosocial resilience. A scoping review is one of many different types of literature reviews. It is similar to an evidence mapping review in that a scoping review employs a systematic search for evidence to respond to specific research questions and maps the evidence; going further than a mapping review, a scoping review typically adds a narrative about the evidence [34]. A scoping review differs from a systematic review in that a scoping review does not evaluate the quality of the evidence found in the literature; further, a scoping review may include both peer-reviewed and grey literature [34,35]. This scoping review includes a review of literature on the topic area as well as a review of the theory and methodology applied to the topic area to better understand the current thinking and empirical approach to the study of climate change and psychosocial health.

This review was conducted between November 2016 to July 2017 for all English articles on the topic area published between 2000 to 2017. Literature included peer-reviewed empirical studies (qualitative, quantitative, and mixed methods) as well as literature reviews, grey literature, and commentaries on the subject matter. The following databases were incorporated in this search: PubMed, Scopus, PsycINFO (Proquest), Cochrane Review, and Google Scholar, using the following keywords (as well as synonyms and related words): “mental health” or “psychosocial” and “climate change”, and “risk” or “impact”, or “adaptation” or “response” or “resilience”. A total of 9079 articles were retrieved. After removing duplicates and scanning the title and abstract of all articles for relevancy, a total of 325 remained. After reviewing the full journal articles, removing irrelevant articles and then searching the reference list of applicable articles, 276 relevant articles were retained.

## 4. Results

Of the 276 relevant articles, the majority (58%) of the articles were literature reviews, including systematic, narrative, and theoretical reviews. Less than 1% of the literature captured was grey literature and only three commentaries were included. The remaining 93 articles were empirical studies; 41% of these articles were quantitative studies, the majority of which were surveys using validated instruments to assess for PTSD, anxiety, and depression. These surveys were administered to understand the psychosocial outcomes related to climate-related extreme weather events—control groups typically included populations who had not experienced extreme weather events first hand. Another 46% were qualitative studies (predominantly interviews and focus groups), and the remainder of the empirical studies (13%) were mixed methods studies that included a combination of interviews and quantitative surveys. A summary of the literature reviewed for this scoping review can be found in the Supplementary Materials.

Importantly, the bulk of empirical literature on climate change and mental health outcomes revealed in this scoping review pertains to direct impacts (like climate-related extreme weather events, e.g., flooding, heatwaves, etc.) at the individual-level from climate-related extreme weather events; as such, the discussion section below emphasizes how to assess the individual mental health outcomes of these direct, climate-related hazards. Notably, there is much less literature on how to assess more chronic climate change related effects (e.g., psychosocial consequences of melting permafrost, sea-level rise, and the overarching awareness of climate change), and less literature on how to assess mental health outcomes of climate change hazards at the group or community level; where possible, we have indicated indicators and measurement tools found in the literature that pertain to chronic climate change related hazards to mental health, as well as indicators and measurement tools that assess climate change hazards beyond individual-level analysis. These gaps in research are acknowledged and future research recommendations are noted in the conclusion and recommendation section.

## 5. Analysis

After reviewing the emergent themes from the scoping review, we asked the following key questions of the current body of knowledge on climate change and mental health as it pertains to CCHVAAs to guide the analysis:

- How can mental health considerations be integrated into CCHVAAs?
- How can the current literature support health authorities to assess the burden of mental illness in relation to climate change?
- How can the current literature support health authorities to assess positive mental health in relation to climate change?
- What would a comprehensive assessment of climate change impacts to mental health look like?
- What are the limitations of integrating mental health indicators into climate change and health assessments?
- What are the opportunities of assessing mental health adaptation opportunities?

These guiding questions helped to reveal the current state of evidence on climate change and mental health, including methods, approaches, documented outcomes, and the authors’ understanding of CCHVA tools and the information health authorities need to be able to address mental health within CCHVAs.

## 6. Discussion

This section provides an overview of key concepts related to the field of climate change and mental health, an exploration of current research methods and approaches in the field, and addresses why the gaps in research on climate change and mental health exist—including an exploration of attributional challenges. Following these topic areas, we provide an overview of key themes that emerged from the scoping review as they pertain to an application of climate change, mental health knowledge and evidence into CCHVAAs.

### 6.1. Defining Health

For climate change and health adaptation assessments to truly capture the health implications of climate change, a holistic conceptualization of health is needed in the planning and implementation of these assessment tools—one that includes both physical and mental health, and that captures affirmative mental and physical health as well as infirmity and disease. Further, we maintain that it is important to understand that mental health is located in the larger domain of psychosocial wellbeing, which links psychological and social wellness. Psychosocial wellbeing is affected by social, spiritual, ecological, historical, and economic circumstances, and is ultimately affected by the social determinants of health.

### 6.2. Current Methods and Approaches

With a specific focus on empirical studies that link climate change and mental health in this scoping review, empirical studies tend to apply either surveys or interview methods to learn about the lived experience of psychosocial distress associated with a changing climate. There are very few studies that assess psychosocial resilience or affirmative mental health outcomes related to a changing climate. Most surveys exploring psychosocial distress rely on self-reported accounts from people who have experienced extreme weather events. These surveys employ a variety of survey tools and scales (that have been validated in most cases) to assess how extreme temperatures or extreme events impact people’s mental health and wellbeing. For example, Albrecht et al. (2007) use the Environmental Distress Scale (EDS) to assess wellbeing in changing environmental conditions [36]. Alderman et al. (2013) use the Kessler 6 (K6) psychological distress scale, the Post-Traumatic Stress Disorder-Civilian checklist (PCL-C), and the Groninger Sleep Quality Scale (GSQS) to assess the psychological impacts of flooding [37]. Eisenman et al. (2015) also used the Kessler 6 (K6) psychological distress scale to understand psychological distress related to the impacts of forest fires in Australia [38]. Crabtree (2013) conducted interviews and administered the General Health Questionnaire (GHQ) to understand psychosocial resilience post-flood [39].

In the study of Inuit in the circumpolar north noted earlier, researchers took a mixed-methods approach that included 72 qualitative interviews and questionnaires [40]. In this same multi-year study, researchers conducted interviews and surveys, as well as photovoice methods, to understand how climate change impacts Inuit in Canada’s northern communities [41]. Photovoice had research participants taking photographs that visually represent how climate change impacts their health and wellbeing [41]. Another approach has been to document emergency room visits for patients checking-in for mental health issues during heat waves or periods of extreme temperatures [42,43,44]. Following the 2013 southern Alberta flood, Sahni et al. (2016) reviewed prescription drug records to document an increase in new prescriptions for anti-anxiety and sleep aid medication following this extreme weather event [45].

These examples illustrate the range of both qualitative, quantitative, and mixed methods approaches used to understand the mental health effects related to a changing climate. Notably absent are methods that simultaneously address both positive and negative psychosocial impacts, a topic that will be addressed later in this discussion.

### 6.3. The Challenges of Attribution

Mental health is understudied in the field of climate change and health in part because of the challenges of attributing particular extreme weather events to climate change, as well as attributing mental health outcomes to climate change related extreme weather. Directional causality of the former has been well established by scholars in the climate change field who note that anthropogenic climate change affects (at the aggregated level) the frequency, magnitude, and intensity of extreme weather [46]. However, attributing causality of a specific event to climate change is challenging. Attributing climate-related extreme weather to mental health outcomes is also challenging because there are few visible indicators of trauma when people are experiencing mental illness, mental problems, or affirmative mental health; as such, it becomes more difficult to establish direct cause and effect relationships [47]. Further, mental problems and mental illness remain stigmatized around the world and people may avoid talking about or seeking mental health care in an effort to avoid stigmatization. Another reason why attribution is challenging is because mental health outcomes may emerge months or years after a traumatic experience, and triggers may not be obvious, even to those experiencing them, nor consciously related to the traumatic experience.

Of note, the bulk of the literature describes three key timeframes for the onset of psychosocial impacts: immediate (hours, days, weeks), mid-range (e.g., six months to a year), and one year and beyond [10,14,48,49,50]. Immediate effects of extreme weather events are referred to as acute trauma and are often framed as normative responses to disaster (e.g., ‘normal reactions to abnormal situations’). This kind of trauma tends to subside once security and safety are established [10]. Mid-range and long-term psychosocial stressors highlighted in the literature tend include anxiety, depression, stress (including PTSD), and drug and alcohol abuse that can occur after an extreme event or related to the awareness of the threats posed by a changing climate [10,11,14]. An additional challenge to address is that the mental health implications of climate change may not be linked to one or more climate-related weather extremes, but rather to the overarching awareness of the climate change problem, which to date has received little attention in the field of psychology and psychiatry [10].

Despite the challenges of attributing climate change to mental health, there are advantages to doing so. Firstly, a better understanding of the true burden of illness associated with climate change can emerge—one that reflects a more holistic conceptualization of health [51]. Identifying climate change as a determinant of mental health may help to reduce stigma. With the knowledge that environmental factors shape mental health, people may be more likely to engage in help-seeking behaviors; further, this knowledge may also encourage environmental stewardship. Locating mental health problems within the broader field of anthropogenic climate change may encourage climate change mitigation behaviors and advocacy for climate action. A robust understanding of the mental health outcomes from climate change—an understanding that includes the affirmative mental health outcomes like post-traumatic growth, altruism, and compassion—can inform the investigation of factors that can enhance or build psychosocial resilience [51].

The extent to which individuals feel capable of taking effective action to reduce climate related risks may play an important role in shaping psychosocial responses to climate change. Some of the psychosocial outcomes that tend to be overlooked in the literature are the complicated and, at times, affirmative mental health effects of responding to a changing climate. Changing climates can stimulate civic action to strengthen climate change mitigation and adaptation outcomes while also encouraging altruism, compassion, and growth. One example of this is the Transition Town movement, a network of citizen-led initiatives in communities around the world that often includes a Heart and Soul group; the Transition Town movement explores the psychosocial impacts of peak oil, climate change, and environmental degradation [52]. Butler et al. (2014) note that engagement in groups like Transition Town provides a space not only for activism, but also improved overall mental health related to collaborative, community-based resilience [53]. Collective action provides communities and individuals with ways to navigate the wicked problems related to a changing climate while strengthening emotional resilience, in addition to building community resilience and hopefully impacting climate change itself. Some examples of co-benefits that support psychosocial health without further contributing to carbon emissions include: walking and biking instead of driving, eating plant-based foods and reducing meat consumption, and engaging in community-based projects focused on environmental stewardship [54].

### 6.4. Integrating Mental Health Indicators into Climate Change and Health Vulnerability and Adaptation Assessments

Like most assessment tools, the basic steps in CCHAAs include framing the assessment, assessing indicators, and monitoring and evaluation. The World Health Organization (WHO) guidance document on CCHVAAs describes these as [55]:

Step 1: Scanning and scope the assessment;
Step 2: Assess vulnerabilities;
Step 3: Assess future climate impacts;
Step 4: Assess adaptation opportunities;
Step 5: Manage and monitor risks through an iterative process, including an evaluation of health harms in other sectors.

While these steps do not actively exclude mental health indicators, mental health tends to be missed in CCHVAAs. The reasons sometimes given for this include: a lack of awareness about mental health and climate change indicators, scarcity of surveillance data, perceived lack of effective mental health care responses, and mental health is seen as less of a pressing concern (in comparison to physical health effects) [56]. Despite this, research demonstrates that mental health effects from any form of disaster far exceed the physical health implications [57]. Further, the multi-clausal pathway linking climate change to mental health developed by Helen Berry and colleagues indicates that there are a number of direct (e.g., extreme weather), indirect (e.g., economic loss due to drought or displacement due to sea-level rise) and overarching (awareness of the climate change problem) pathways from which mental health may be affected by climate change, suggesting there are in fact numerous climate-related indicators affecting mental health [21].

Existing guidance frameworks that support the evaluation of the mental health implications of policies, programs, and strategies include the Wellesley Institute’s primer report entitled Mental Well-Being Impact Assessment, and the UK’s Mental Well-being Impact Assessment toolkit [58,59]. While these frameworks can be integrated into existing CCHVAA tools, there are no known applications to date. Both frameworks incorporate a health equity lens and take a holistic approach to mental health, wherein mental health is seen to include mental well-being as well as mental problems and illness, and the objectives of these mental well-being impact assessment frameworks is to minimize mental health problems and issues and maximize mental wellbeing. These assessment frameworks support the analysis of the mental health consequences of a specific policy, project, or service; however, they do not provide guidance on specific measurement or monitoring techniques.

### 6.5. Assessing the Burden of Mental Illness in Relation to Climate Change

To support the monitoring and surveillance of mental health indicators in a changing climate, specific strategies are needed. There are a number of disaster mental health tools that can be repurposed to address climate-related disasters. The Inter-Agency Standing Committee (IASC), developed in partnership with the WHO, created a set of guidelines on mental health and psychosocial support in emergency settings [60]. The IASC guidelines recommend epidemiological surveys of mental disorders and distress of the general population following extreme weather events that take into consideration indicators of risk (e.g., marginalized status) and protective factors (e.g., access to mental health care). One potential toolkit that can be administered in this regard is the Disaster Psychological Assessment and Surveillance Toolkit (Disaster-PAST), an open-access resource that provides psychosocial assessment and surveillance tools to improve disaster preparedness and response and to enhance community recovery. This toolkit was developed by the Louisiana State University Health Sciences Center Department of Psychiatry and the Louisiana State Department of Health and Hospitals Office of Behavioral Health [61] following Hurricane Katrina. Disaster-PAST provides a framework for three distinct post-disaster screening phases: the immediate screening phase (1–60 days post-event); the recovery screening phase (60 days–1 year post-event); and the extended screening phase (over one year post-event) [61]. Some of the validated scales used in this toolkit include the Generalized Anxiety Disorder Scale (GAD-7), the Center for Epidemiologic Studies Depression Scale (CES-D), and the Post-Traumatic Stress Checklist (PCL-C) [61]. One of the benefits of Disaster-PAST is that it provides distinct time frames to assess the state of mental health following an extreme event, taking into account that the timing and triggers related to a changing climate vary. A drawback of this toolkit, however, is that it focuses primarily on mental health illness and mental problems and overlooks the positive mental health impacts of climate-related extreme weather events.

It is important to highlight that while epidemiological surveys can provide useful information on distress, coping, and recovery, there is a tendency for these to exaggerate mental health disorders, especially if they are administered soon after a disaster because people may self-report mental health disorders that are not actually pathological but rather normal responses to abnormal situations. Also of note, many survey tools have not been validated in cultural contexts outside of the western world, so their use may be geographically and contextually limited. As a result of these limitations, IASC recommends that special consideration be given to validating survey instruments in specific cultural contexts and that assessment indicators include severe mental health problems (e.g., suicidal tendencies, dangerous behaviors and behaviors that impair daily functioning) to better gauge the state of mental health beyond mental disorders that may not be pathological. Further, we argue that it is equally important to include positive mental health outcomes to better gauge the full scope of psychosocial outcomes.

### 6.6. Assessing Positive Mental Health in Relation to Climate Change

In “A Paradise Built in Hell” (2009), Rebecca Solnit aptly describes the complicated psychosocial consequences that can arise during and after an extreme event as:

“that sense of immersion in the moment and solidarity with others caused by the rupture in everyday life, an emotion graver than happiness but deeply positive. We don’t even have a language for this emotion, in which the wonderful comes wrapped in the terrible, joy in sorrow, courage in fear. We cannot welcome disaster, but we can value the responses, both practical and psychological”.

[62]

What Solnit describes is something akin to, but perhaps more than, ‘coming to our senses’. She describes how habitual norms of respectful distance, busyness, and isolation are suspended in disasters in ways that allow for deep connection, a sense of contribution and aliveness. Given the tragedy of disaster, these conflicting emotions are often unsettling.

In “Climate Change and Human Well-being”, Weissbecker (2011), explores a range of psychosocial consequences of climate change. This range includes the propensity for mental health problems and illness as well as empathy, compassion, altruism, emotional resilience, and post-traumatic growth (PTG) [22].

Understanding the positive mental health outcomes following a climate-related event is pertinent because this information can provide insights into the complex, multidimensional effects of a changing climate on human health and wellbeing, and this understanding may also support climate change and health adaptation efforts [51]. One suggested approach is to assess post-traumatic growth (PTG) after a climate change-related extreme weather event. Tedeschi and Calhoun (1995), who coined the term, describe it as “significant beneficial changes in cognitive and emotional life beyond levels of adaptation, psychological functioning, or life awareness that occur in the aftermath of psychological traumas that challenge previously existing assumptions about self, others, and the future” [63]. Some scholars define PTG as a specific form of resilience [64]. Resilience in the mental health literature is often conceptualized as an outcome of the coping process, as an adaptive process, or as a multifaceted construct that encompasses both process and outcome [63]. Some scholars contend that PTG is distinct from resilience, because PTG is a transformative [65]. Regardless, PTG is considered an affirmative, psychosocial response to trauma, and this response ought to be considered in an investigation of climate change hazards and their effects on mental health.

As with PTSD, scholars note that PTG tends only to occur in the context of significant traumatic stressors [66]. Traditionally PTG is measured via interview questions or via surveys. Interview questions often probe participants to “to identify the ways in which their lives had changed as a result of their trauma” [67], or to probe how participants perceive growth after experiencing trauma [67]. Surveys are often conducted using one of the three psychometrically validated scales: Stress-Related Growth Scale (SRGS), the Post-Traumatic Growth Inventory (PTGI), and the Benefit Finding Scale (BFS) [67]. Researchers often verify self-reported survey and interview outcomes by conducting interviews or surveys with people who are close to respondents or by using control and comparison groups [67]. We note that several questions remain, including whether the concept of PTG is only culturally relevant to the western world, and just like measuring and monitoring mental health illness or problems after a traumatic event, what is the best timing to assess for PTG.

### 6.7. Towards a Comprehensive Assessment of Climate Change Impacts to Mental Health

We have highlighted several measurement tools that can support the monitoring and surveillance of climate change impacts to mental health. What is needed is a comprehensive approach to the measurement and monitoring of the multi-clausal pathways of climate impacts to mental health and an evaluation of the resources that support mental health in a changing climate. Specifically, we argue that monitoring and surveillance strategies that support health officials’ understanding of the full spectrum of climate impacts to mental health are needed. These strategies ought to include direct impacts from extreme weather events, the indirect impacts from climate hazards (e.g., drought, sea-level rise) that affect economic and social security and subsequently have mental health implications, as well as indicators that address the overarching awareness of the climate change problem that affects psychosocial wellbeing. The last point is particularly resonant given how much we are now being informed by the media that this is something we should be worried about. Further, a robust understanding of availability and access to mental wellness resources and interventions is needed to better understand health adaptations to our changing climate.

Table 1 outlines measurement and monitoring techniques that can be incorporated into Step 2 (assessing vulnerabilities) of existing CCHVAAs. The climate hazards that are listed in the first column are the climate-related hazards that are most known to affect mental health, as indicated in the literature. The ‘Populations of Concern’ column lists population groups who have been found to be most affected by each hazard type. Importantly, the population groups mentioned in this table are not mutually exclusive groups, in so far as people may belong to more than one social group. The column entitled ‘Potential Mental Health Outcomes’ highlights the possible mental health consequences of specific climate hazards. These potential outcomes are not meant to be an exhaustive list, but rather a list of some of the mental health outcomes found within the literature on climate change and extreme weather. The last column highlights some ways in which mental health outcomes from a changing climate can be surveyed and monitored. These measurement tools have been chosen based on their application in prior studies that address mental health surveillance and monitoring related to climate change and/or extreme weather.

Once information about the mental health vulnerabilities associated with climate change has been incorporated into Step 2, health authorities are then able to proceed with Step 3 (assessing future climate impacts). Predicting the mental health impacts from climate change is similar to predicting the physical health impacts; predictions are based upon an analysis of past health trends related to climate change and modelling future climate changes. Most climate change projections are based upon predictions of greenhouse gas (GHG) emissions, social (lifestyle, energy and technology use), environmental (land use patterns), and demographic factors (population size). The most widely used projections are the representative concentration pathways (RCPs) as described in the Intergovernmental Panel on Climate Change (IPCC) fifth assessment (IPCC, 2014). The RCPs incorporate “four different 21st century pathways of GHG emissions and atmospheric concentrations, air pollutant emissions and land use. The RCPs include a stringent mitigation scenario (RCP2.6), two intermediate scenarios (RCP4.5 and RCP6.0) and one scenario with higher GHG emissions (RCP8.5). Scenarios without additional efforts to constrain emissions (’baseline scenarios’) lead to pathways ranging between RCP6.0 and RCP8.5” [68]. Information from the RCPs, combined with localized weather and climate data, and health vulnerabilities assessed in Step 2, all support Step 3 of climate change and health assessments.

### 6.8. Limitations of Current Efforts to Integrate Mental Health Indicators into Climate Change and Health Assessments

A few limitations with the table above are important to highlight here. Firstly, there are fewer empirical studies in the scoping review that reveal indicators and measurement tools related to chronic climate change-related hazards (e.g., sea-level rise, melting permafrost, and climate change writ large) compared to the empirical evidence related to direct climate change hazards (e.g., extreme heat, extreme weather events). As such, the indicators and measurement tools for direct climate hazards appear more robust. Secondly, empirical studies in the scoping review tend to rely upon individual-level indicators of mental health consequences of climate change; as such, there are fewer measurement tools that can be applied at the group or community level. Notably, this may prove to be more labor and cost intensive for health authorities; one suggestion would be to partner with academic institutions for this type of individual-level analysis. Thirdly, there are few recommendations in the literature on the timelines for monitoring mental health outcomes from climate hazards via emergency department visits, medical records, and syndromic surveillance. With the exception of heat waves—where the time frame to monitor emergency departments is directly linked to the duration of the heat wave—there are no clear timelines for monitoring medical records and emergency department visits when it comes to mental health outcomes related to climate-related extreme weather hazards or more chronic climate-related hazards (e.g., sea-level, melting permafrost, knowledge of climate change writ large). As noted above in the section on attributional challenges, mental health outcomes from a climate-related hazard may emerge long after a hazard has passed; thus, the timeframe to study the mental health outcomes remains a challenge. Finally, there is currently a lack of available guidance on how to accurately monitor International Classification of Disease (ICD) codes related to broad mental health indicators, like depression for example.

The objective of providing Table 1 is to provide health authorities with initial guidance to support the integration of mental health into CCHVAAs based on the current state of evidence on climate change and mental health. This table is not meant to be a prescriptive application but rather an iterative template to support the ongoing measurement and monitoring of the mental health consequences of climate change.

### 6.9. Assessing Mental Health Adaptation Opportunities

To support health authorities with the mental health considerations of Step 4 (assessing adaptation opportunities), this section provides some guidance based on recommendations by IASC. This section concludes with an example of research methods based on IASC recommendations. The IASC guidelines recommend mapping and monitoring psychosocial resources and skills within the community affected by a disaster and mapping the interdisciplinary relationships that can enhance or improve the psychosocial response [60]. This type of surveillance is particularly useful to understand the current breadth and depth of psychosocial resources to support communities before, during, and after a climate-related event; further, this information is useful for policy and program planning as it can reveal skill and resource gaps, providing critical information to support mental health adaptation to a changing climate.

Taking note of this recommended approach, the lead author (Hayes) is conducting her doctoral research on psychosocial adaptation to a changing climate. In this research, she investigates and maps the resources (programs, policies, support networks, services, etc.) available to support the long-term mental health and wellbeing of people in the town of High River, Alberta who experienced the 2013 super flood. Prior to 2001, southern Alberta (where High River is located) was exposed to frequent drought; since 2005, there has been an increase in extreme precipitation and flooding events, the most notable occurring in 2013 that resulted in four deaths and a declared state of emergency where the entire population of High River was displaced [69]. High River, like many communities around the globe, is experiencing the health effects of climate-related extreme weather events. The methods outlined below can be applied, in part or in full, in other communities to support an assessment and evaluation of mental health care capacity in a changing climate.

The research in High River begins with a rapid vulnerability and adaptation assessment that scans and scopes the current and future climate change impacts to mental health in High River and locates key resources that support mental health and wellbeing before, during, and after climate-related extreme weather events. Following this, key informant interviews are conducted with municipal government representatives, public health officials, emergency response members, and mental health care providers to understand the mental health response and response capacity before, during, and after the flood. The 2013 flood acts as the primary focusing event for research participants. The next phase of the research includes a critical discourse analysis of policy documents and media articles that outline responses in High River to the psychosocial health consequences of climate change. The researcher is mapping psychosocial assets in the community; this will be achieved by conducting focus groups with front-line public health workers to explore the formal and informal psychosocial health care resources available to people in High River. Formal health care resources may include things like access to therapies, counselling, and pharmacotherapeutics; informal mental health care resources may include faith-based institutions and community support groups. The inclusion of informal mental health care is important in this research because there are many psychosocial adaptation opportunities that lay outside formal mental health care. For example, Joanna Macy’s “Work that Reconnects” provides practices and networks to support people to confront the psychological and spiritual effects of environmental degradation and awaken to renewed hope and active engagement to address climate change implications to wellbeing [70]. Carolyn Baker’s “Navigating the Coming Chaos” is a toolkit designed to support people as they consciously awaken to their climate reality through emotional and spiritual resiliency [71]. By mapping both the formal and informal (or non-traditional) mental health care opportunities, we are better able to capture and assess the full spectrum of psychosocial adaptation opportunities.

The last phase of research consists of face-to-face interviews with a sample of marginalized community members to explore their experiences with psychosocial response interventions. The aim is to understand the psychosocial resource supports from the perspective of the residents who are most vulnerable to the impacts of a changing climate based on their social status, and to acquire an understanding of the accessibility of mental health resources (formal and informal) to those who are at highest risk. To our knowledge, this is the first comprehensive research project that investigates psychosocial resources to support the mental health consequences of climate change in this way, from a variety of resident and mental health professional perspectives. Results are anticipated to be available in late 2019.

## 7. Conclusions

In the aftermath of Hurricane Katrina and Hurricane Sandy, public health officials in the U.S advocated for the application of vulnerability and adaptation assessments to support and enhance response interventions to extreme weather events. Schmeltz et al. (2013) notes that “vulnerability assessments provide the evidence and build the political will necessary to design, fund, and implement adaptive measures that reduce the costs of interventions and responses, protect vulnerable populations, and save lives during extreme weather events.” [72]. The integration of mental health measurement and monitoring strategies within CCHVAAs can support decision makers with evidence-based information on the psychosocial consequences of a changing climate, and therefore support better planning, preparation for, and adaption to the mental health implications of climate change.

### Recommendations

As noted, the majority (58%) of articles in this scoping review on mental health and climate change comes in the form of literature reviews. With that said, however, this scoping review identified a number of peer-reviewed, empirical studies on the topic area (93 articles). The current state of evidence in this field provides health authorities with a basis of knowledge to support an exploration of mental health and climate change in CCHVAAs. While the field of climate change and mental health is growing, it is noted that additional empirical studies on climate change and mental health are needed to advance evidence and knowledge in this field of study and to strengthen decision-making regarding climate change and mental health adaptation. In particular, what is needed is empirical research that supports surveillance and monitoring of the psychosocial consequences of climate change that explores:

- the mental health consequences of indirect and chronic climate hazards (like pervasive drought, sea-level rise, and melting permafrost; and loss of community when entire towns are affected by climate-related events);
- the overarching mental health implications related to the knowledge of climate change writ large (e.g., amongst climate change researchers, environmental activists, and others immersed in the science and work associated with climate change and environmental degradation);
- affirmative mental health outcomes related to community resilience in the aftermath of extreme weather events (a.k.a. post-traumatic growth);
- the application of measurement and monitoring tools that investigate group and community-level mental health outcomes related to climate hazards (e.g., community resilience, sense of community, mutual aid, etc.); and,
- research that addresses the timing and triggers of mental health outcomes related to climate change hazards (e.g., initial versus delayed impacts, both positive and negative).

A further recommendation in this research domain includes: guidance for health authorities on how to accurately monitor International Classification of Disease (ICD) codes related to broad mental health indicators.

The opportunities to explore the risks, impacts, and response interventions related to climate change and mental health have policy implications that support climate change mitigation and adaptation as well as population-level mental health. One of the chief aims of documenting the mental health implications of climate change is to support and enhance the sustainability and resilience of mental health systems—a topic area that tends to be absent from the discourse on the resilience of broader health systems. Through an evidence-based review of the potentially long-term mental health consequences of climate change, decision makers have a better understanding of where and how to invest in mental health infrastructure and resources. Additionally, assessments that investigate and map formal and informal mental health interventions can provide decision makers and communities with a robust understanding of all resources currently in place, or needed, to address climate change-related distress, enhance affirmative mental health, and support psychosocial resilience.

## Figures and Tables

**Table 1:** Monitoring and Measuring the Climate Change Impacts to Mental Health.

Climate Hazard	Populations of Concern	Potential Mental Health Outcomes	Indicators and Measurement Tools
Extreme Heat	People with pre-existing mental health conditions. People taking psychotropic medications that affect thermoregulation. Elderly (who have poor thermoregulation). People with substance abuse problems People living in urban heat islands Urban poor without access to air conditioning Those living on the street Outdoor laborers	Exacerbated mood or behavioral disorders Violence Aggression Suicide Other	Monitor emergency department visits after heat waves for an increase in patients reporting mood or behavioral disorders. Monitoring mortality statistics following extreme heat events—look for co-morbidities related to mental health and incidents of suicide. Interviews or questionnaires with people who experienced heat waves or extreme heat events to ask about their mental health in relation to heat events. Review of police records following extreme heat events to monitor elevated incidents of violence or aggression.
Extreme Weather Event (flood, hurricane, drought, mudslides, etc.)	Gender (Female) Sex (Female, particularly pregnant women) Age (children, infants, seniors) Race and ethnicity (non-Caucasian, non-white) Immigrants People with pre-existing health conditions People with low-socioeconomic status The under and non-insured (health care and home insurance) The under-housed and homeless Outdoor laborers First responders	Post-traumatic stress disorder (PTSD) Depression (including major depressive disorders) Anxiety Suicidal ideation Aggression Substance abuse and addiction Violence Survivor guilt Vicarious trauma Altruism Compassion Post-traumatic growth Other	Surveys Self-report surveys of general health. Consider using: - General Health Questionnaire (GHQ) Self-report surveys of mental illness and mental problems. Consider using any, or a combination of: - Disaster-PAST [61]; the Generalized Anxiety Disorder Scale (GAD-7); the Post-Traumatic Stress Disorder Checklist (PCL); The Center for Epidemiologic Studies Depression Scale (CES-D); the Kessler Psychological Distress Scale (K6) Self-report surveys of affirmative mental health. Consider using: - Stress-Related Growth Scale (SRGS); Post-Traumatic Growth Index (PTGI); Benefit Finding Scale (BFS) Patient Records Monitor emergency department visits after extreme weather events for an increase in patients reporting mental health problems or illness. Review of new prescription use for mental health and behavioral disorders after an extreme weather event Interviews - Interviews with primary care physicians and mental health care providers about any surges in patients reporting mental health issues following extreme weather events. - Interviews with people who experienced an extreme weather event about their perceptions regarding their mental health related to the extreme weather event.
Vector-borne disease (VBD) (e.g., Lyme Disease, West Nile Virus, Ticks)	Homeless People with pre-existing mental health conditions Outdoor workers	VBD disease (particularly: Lyme Disease or West Nile Virus) and compounded mental health problems (e.g., cognitive or neurological impairment, behavioral disorders)	Interviews or questionnaires with patients who have been diagnosed with VBDs to ask about perceptions of their mental health. Interviews with primary care physicians and mental health care providers about any mental health co-morbidities for patients diagnosed with VBDs.
Sea-Level Rise or Melting Permafrost	People who work or live near the ocean (sea-level rise) or in the arctic Outdoor laborers Indigenous people	Anxiety, worry, or fear of displacement Anxiety, worry, or fear of job loss Loss of place (grief, solace)	Interviews or questionnaires with residents who have or are experiencing sea-level rise or prolonged drought in their communities. Interview questions may focus on the mental health implications of: displacement, job loss associated with sea-level rise, infrastructure damage, agricultural or resource loss and resource scarcity, food and water safety and security.
Climate Change writ large (i.e., awareness of climate change threats to human and planetary health and survival)	People at greater risk from and exposure to climate change Researchers investigating climate change Environmental and climate change activists Environmental studies students Outdoor recreationalists Indigenous peoples	Anxiety Worry Stress Fear	Interviews or questionnaires with people who experience concern, anxiety, worry, related to awareness of climate change threats. The Generalized Anxiety Disorder Scale (GAD-7)

## Supplementary Materials

The following are available online at http://www.mdpi.com/1660-4601/15/9/1806/s1.

## References

[1] Costello A., Abbas M., Allen A., Ball S., Bell S., Bellamy R., Friel S., Groce N., Johnson A., Kett M. (2009). Managing the health effects of climate change. Lancet.

[2] Berry P., Clarke K.-L., Parker S., Warren F.J., Lemmen D.S. (2014). Human health. Canada in a Changing Climate: Sector Perspectives on Impacts and Adaptation.

[3] Wilson K. (2003). Therapeutic landscapes and First Nations peoples: An exploration of culture, health and place. Health Place.

[4] Kumar U. (2016). The Routledge International Handbook of Psychosocial Resilience.

[5] Watts N., Amann M., Ayeb-Karlsson S., Belesova K., Bouley T., Boykoff M., Byass P., Cai W., Campbell-Lendrum D., Chambers J. (2018). The Lancet Countdown on health and climate change: From 25 years of inaction to a global transformation for public health. Lancet.

[6] McMichael A. (2017). Climate Change and the Health of Nations.

[7] Crimmins A., Balbus J., Gamble J.L., Beard C.B., Bell E., Dodgen D., Eisen R.J., Fann N., Hawkins M.D., Ziska L. (2016). The Impacts of Climate Change on Human Health in the United States: A Scientific Assessment.

[8] Watts N., Adger W.N., Agnolucci P., Blackstock J., Byass P., Cai W., Chaytor S., Colbourn T., Collins M., Cooper A. (2015). Health and climate change: Policy responses to protect public health (The Lancet Commission). Lancet.

[9] Luber G., Lemery J. (2015). Global Climate Change and Human Health: From Science to Practice.

[10] Fritze J.G., Blashki G.A., Burke S., Wiseman J. (2008). Hope, despair and transformation: Climate change and the promotion of mental health and wellbeing. Int. J. Ment. Health Syst..

[11] Dodgen D., Donato D., Kelly N., La Greca A., Morganstein J., Reser J., Schweitzer S., Shimamoto M.M., Tart K.T., Ursano R. (2016). Mental health and well-being. The Impacts of Climate Change on Human Health in the United States: A Scientific Assessment.

[12] Cusack L., de Crespigny C., Athanasos P. (2011). Heatwaves and their impact on people with alcohol, drug and mental health conditions: A discussion paper on clinical practice considerations. J. Adv. Nurs..

[13] Page L.A., Hajat S., Kovats R.S., Howard L.M. (2012). Temperature-related deaths in people with psychosis, dementia and substance misuse. Br. J. Psychiatry.

[14] Clayton S., Manning C.M., Krygsman K., Speiser M. (2017). Mental Health and Our Changing Climate: Impacts, Implications, and Guidance.

[15] Fernandez A., Black J., Jones M., Wilson L., Salvador-Carulla L., Astell-Burt T., Black D. (2015). Flooding and mental health: A systematic mapping review. PLoS ONE.

[16] Neria Y., Shultz J.M. (2012). Mental health effects of Hurricane Sandy: Characteristics, potential aftermath, and response. JAMA.

[17] Bryant R.A., Waters E., Gibbs L., Gallagher H.C., Pattison P., Lusher D., MacDougall C., Harms L., Block K., Sinnott V. (2014). Psychological outcomes following the Victorian Black Saturday bushfires. Aust. N. Z. J. Psychiatry.

[18] Gleick P.H. (2014). Water, drought, climate change, and conflict in Syria. Weather Clim. Soc..

[19] Cunsolo Willox A.C., Harper S.L., Ford J.D., Edge V.L., Landman K., Houle K., Blake S., Wolfrey C. (2013). Climate change and mental health: An exploratory case study from Rigolet, Nunatsiavut, Canada. Clim. Chang..

[20] Cunsolo Willox A.C., Stephenson E., Allen J., Bourque F., Drossos A., Elgaroy S., Kral M.J., Mauro I., Moses J., Wexler L. (2014). Examining relationships between climate change and mental health in the Circumpolar North. Reg. Environ. Chang..

[21] Berry H.L., Bowen K., Kjellstrom T. (2010). Climate change and mental health: A causal pathways framework. Int. J. Public Health.

[22] Weissbecker I. (2011). Climate Change and Human Well-Being: Global Challenges and Opportunities.

[23] Berry H. (2009). Pearl in the oyster: Climate change as a mental health opportunity. Australas. Psychiatry.

[24] Berry H.L., Shipley M. (2009). Longing to Belong: Personal Social Capital and Psychological Distress in an Australian Coastal Region.

[25] Bowles D.C. (2015). Climate change and health adaptation: Consequences for indigenous physical and mental health. Ann. Glob. Health.

[26] Green D., Minchin L. (2014). Living on climate-changed country: Indigenous health, well-being and climate change in remote Australian communities. EcoHealth.

[27] Bardsley D.K., Wiseman N.D. (2012). Climate change vulnerability and social development for remote indigenous communities of South Australia. Glob. Environ. Chang..

[28] Durkalec A., Furgal C., Skinner M.W., Sheldon T. (2015). Climate change influences on environment as a determinant of Indigenous health: Relationships to place, sea ice, and health in an Inuit community. Soc. Sci. Med..

[29] Duncan K., Guidottti T., Cheng W., Naidoo K., Gibson G., Kalkstein L., Last J., Maxwell B., Mayer N., Street R. (1997). Health sector in Canada country study: Impacts and Adaptations. The Canada Country Study: Climate Impacts and Adaptation, National Summary for Policy Makers.

[30] Natural Resources Canada (NRCAN) Canada in a Changing Climate: Science and Information Products. https://www.nrcan.gc.ca/environment/impacts-adaptation/19922.

[31] Berry P. (2017). Climate change and the health of Canadians: Impacts and adaptation in northern Ontario. Proceeding of the Northwestern Health Unit: Climate Change and our Health.

[32] U.S. Global Change Research Program (USGCRP) Legal Mandate. https://www.globalchange.gov/about/legal-mandate.

[33] United Kingdom Government Climate Change Act 2008. http://www.legislation.gov.uk/ukpga/2008/27/contents.

[34] Dijkers M. (2015). What is a scoping review. KT Update.

[35] Peterson J., Pearce P.F., Ferguson L.A., Langford C.A. (2017). Understanding scoping reviews: Definition, purpose, and process. J. Am. Assoc. Nurse Pract..

[36] Albrecht G., Sartore G.M., Connor L., Higginbotham N., Freeman S., Kelly B., Stain H., Tonna A., Pollard G. (2007). Solastalgia: The distress caused by environmental change. Australas. Psychiatry.

[37] Alderman K., Turner L.R., Tong S. (2013). Assessment of the health impacts of the 2011 summer floods in Brisbane. Disaster Med. Public Health Prep..

[38] Eisenman D., McCaffrey S., Donatello I., Marshal G. (2015). An ecosystems and vulnerable populations perspective on solastalgia and psychological distress after a wildfire. EcoHealth.

[39] Crabtree A. (2013). Questioning psychosocial resilience after flooding and the consequences for disaster risk reduction. Soc. Indic. Res..

[40] Cunsolo Willox A.C., Harper S.L., Ford J.D., Landman K., Houle K., Edge V.L. (2012). “From this place and of this place”: Climate change, sense of place, and health in Nunatsiavut, Canada. Soc. Sci. Med..

[41] Harper S.L., Edge V.L., Ford J., Willox A.C., Wood M., McEwen S.A., Namanya D.B. (2015). Climate-sensitive health priorities in Nunatsiavut, Canada. BMC Public Health.

[42] Vida S., Durocher M., Ouarda T.B., Gosselin P. (2012). Relationship between ambient temperature and humidity and visits to mental health emergency departments in Quebec. Psychiatr. Serv..

[43] Wang X., Lavigne E., Ouellette-kuntz H., Chen B.E. (2014). Acute impacts of extreme temperature exposure on emergency room admissions related to mental and behavior disorders in Toronto, Canada. J. Affect. Disord..

[44] Trang P.M., Rocklöv J., Giang K.B., Kullgren G., Nilsson M. (2016). Heatwaves and hospital admissions for mental disorders in Northern Vietnam. PLoS ONE.

[45] Sahni V., Scott A.N., Beliveau M., Varughese M., Dover D.C., Talbot J. (2016). Public health surveillance response following the southern Alberta floods, 2013. Can. J. Public Health.

[46] Parry M.L., Canziani O.F., Palutikof J.P., van der Linden P.J., Hanson C.E. (2007). Contribution of Working Group II to the Fourth Assessment Report of the Intergovernmental Panel on Climate Change.

[47] Watts N., Adger W.N., Ayeb-Karlsson S., Bai Y., Byass P., Campbell-Lendrum D., Depoux A. (2017). The Lancet Countdown: Tracking progress on health and climate change. Lancet.

[48] North C.S., Pfefferbaum B. (2013). Mental health response to community disasters: A systematic review. JAMA.

[49] Anderson H., Brown C., Cameron L.L., Christenson M., Conlon K.C., Dorevitch S., Walker R. (2017). Climate and Health Intervention Assessment: Evidence on Public Health Interventions to Prevent the Negative Health Effects of Climate Change; Climate and Health Technical Report Series; Climate and Health Program, Centers for Disease Control and Prevention, BRACE Midwest and Southeast Community of Practice. https://www.cdc.gov/climateandhealth/docs/ClimateAndHealthInterventionAssessment_508.pdf.

[50] Tunstall S., Tapsell S., Green C., Floyd P., George C. (2006). The health effects of flooding: Social research results from England and Wales. J. Water Health.

[51] Hayes K., Blashki G., Wiseman J., Burke S., Reifels L. (2018). Climate change and mental health: Risks, impacts and priority actions. Int. J. Ment. Health Syst..

[52] Hopkins R. (2008). The Transition Handbook: From Oil Dependency to Local Resilience.

[53] Butler C.D., Bowles D.C., McIver L., Page L., Butler C.D. (2014). Mental health, cognition and the challenge of climate change. Climate Change and Global Health.

[54] Koger S.M., Leslie K.E., Hayes E.D. (2011). Climate change: Psychological solutions and strategies for change. Ecopsychology.

[55] World Health Organization Protecting Health from Climate Change: Vulnerability and Adaptation Assessment. http://www.who.int/globalchange/publications/vulnerability-adaptation/en/.

[56] Patel V., Saxena S., Frankish H., Boyce N. (2016). Sustainable development and global mental health—A Lancet Commission. Lancet.

[57] Links J. (2017). Predicting Community Resilience and Recovery after a Disaster. https://blogs.cdc.gov/publichealthmatters/2017/08/predicting-community-resilience-and-recovery-after-a-disaster/.

[58] Lalani N. Mental Well-Being Impact Assessment: A Primer. http://www.wellesleyinstitute.com/wpcontent/uploads/2011/02/MWIA_Lalani.pdf.

[59] Cooke A., Friedli L., Coggins T., Edmonds N., Michaelson J., O’Hara K., Snowden L., Stanseld J., Steuer N., Scott-Samuel A. National Mental Wellbeing Impact Assessment Collaborative. https://healthycampuses.ca/wp-content/uploads/2014/07/MentalWellbeingImpactAssessmentAtoolkitforwellbe-1.pdf.

[60] Inter-Agency Standing Committee (IASC) (2007). IASC Guidelines on Mental Health and Psychosocial Support in Emergency Settings.

[61] Ciccone A. (2012). Disaster Psychosocial Assessment and Surveillance Toolkit (Disaster-Past): Methods to Enhance Disaster Preparedness, Response and Recovery.

[62] Solnit R. (2009). A Paradise Built in Hell: The Extraordinary Communities That Arise in Disaster.

[63] Paton D., Calhoun L.G., Tedeschi R.G. (2006). Posttraumatic growth in disaster and emergency work. Handbook of Posttraumatic Growth: Research and Practice.

[64] Lepore S.J., Revenson T.A., Calhoun L.G., Tedeschi R.G. (2006). Resilience and posttraumatic growth: Recovery, resistance, and reconfiguration. Handbook of Posttraumatic Growth: Research and Practice.

[65] Tedeschi R.G., Calhoun L.G. (2004). Posttraumatic growth: Conceptual foundations and empirical evidence. Psychol. Inq..

[66] Ramsay T., Manderson L., Weissbecker I. (2011). Resilience, spirituality and posttraumatic growth: Reshaping the effects of climate change. Climate Change and Human Well-Being: Global Challenges and Opportunities.

[67] Park C.L., Lechner S.C., Calhoun L.G., Tedeschi R.G. (2006). Measurement issues in assessing growth following stressful life experiences. Handbook of Posttraumatic Growth: Research and Practice.

[68] Pacharui R.K., Meyer L.A. (2014). Climate Change 2014: Synthesis Report, Contribution of Working Groups I, II and III to the Fifth Assessment Report of the Intergovernmental Panel on Climate.

[69] Public Safety Canada The Canadian Disaster Database. http://www.publicsafety.gc.ca/cnt/rsrcs/cndn-dsstr-dtbs/index-eng.aspx.

[70] Macy J. Work That Reconnects Network. https://workthatreconnects.org.

[71] Baker C. (2011). Navigating the Coming Chaos: A Handbook for Inner Transition.

[72] Schmeltz M.T., González S.K., Fuentes L., Kwan A., Ortega-Williams A., Cowan L.P. (2013). Lessons from hurricane sandy: A Community response in Brooklyn, New York. J. Urban Health.

