# Supplementary material for: Addressing Mental Health in a Changing Climate: Incorporating Mental Health Indicators into Climate Change and Health Vulnerability and Adaptation Assessments
Source: Int J Environ Res Public Health. 2018 Aug 22;15(9):1806. doi: 10.3390/ijerph15091806 (PMC6164893; doi:10.3390/ijerph15091806)
Supplement: Supplementary file 1 [file ijerph-15-01806-s001.pdf]

Supplementary Material

Table S1. Summary of Articles Included in Review

Article #	Article Citation	Country	Literature Type	Study Design (where applicable)	Participants	Outcomes Measured
1.	Adger et al. (2011)	Maldives and Canada	Empirical Research Article	Qualitative Case Study	Case study of Pacific Islanders of Atolls and Inuit in the Canadian Arctic	Relationship to place (emotional, symbolic, environmental, spiritual)
2.	Ager et at. (2015)	International	Empirical Research Article	Qualitative interviews and document analysis	Humanitarian and Non-Governmental Organizations	Emergency response (including local faith-based community response), disaster risk reduction, transitional and sustainable solutions
3.	Agnew et al. (2012)	Global	Literature Review – Theoretical review (of crime and climate change)	N/A	N/A	N/A
4.	Aiena et al. (2015)	USA	Empirical Research Article	Quantitative survey (Measuring resilience with RS 14)	Adults post-gulf oil spill and university students	Resilience related to environmental degradation
5.	Aiena, et al. (2016)	USA	Empirical Research Article	Quantitative survey (Measuring resilience, meaning and PTSD)	Mississippi coastal residents (perceived effect group and non-perceived effect group)	Perceptions of meaning, resilience, and Post-Traumatic Stress Disorder related to

Article #	Article Citation	Country	Literature Type	Study Design (where applicable)	Participants	Outcomes Measured
						environmental degradation
6.	Aitsi-Selmi et al. (2015)	International	Literature Review – Narrative Review (Health and wellbeing post disaster)	N/A	N/A	N/A
7.	Ajibade et al. (2015)	Nigeria	Empirical Research Article	Quantitative Survey (13 Item Additive Scale – questions pertained to whether or not respondents had experienced: damage to properties, impacts to individual health, restricted movement, infrastructure damage, and loss of lives	1003 flood impacted Nigerians in Lagos	Flood impacts based on a bio-psychosocial model of socio-economic, demographic, behavioural and environmental factors
8.	Ajibade et al. (2013)	Nigeria	Empirical Research Article	Mixed methods (Interviews, survey and focus groups)	Women in Lagos, Nigeria who experienced flash floods; 36 interviews, a survey (n = 453) and 6 focus groups	Vulnerability and resilience among flooded women in Lagos, Nigeria

Article #	Article Citation	Country	Literature Type	Study Design (where applicable)	Participants	Outcomes Measured
9.	Alberini et al. (2006).	International	Empirical Research Article	Quantitative – survey (conjoint choice questions about adaptive capacity to climate change)	100 Climate change (climatologists), public health professionals, and emergency response professionals	Perceptions about adaptive capacity to climate change
10.	Albrecht et al. (2007)	Australia	Empirical Research Article	Mixed Methods (qualitative interviews; ethnographic field work, surveys using the Environmental Distress ED scale)	Interviews with 60 people living in Upper Hunter (region with persistent drought and mining).<br><br>Ethnographic field work and surveys with residents of Upper Hunter region	Distress related to environmental change (drought and mining)
11.	Albrecht (2011)	Globally - examples mainly based in Australia; also refers to Inuit	Grey Literature (chronic climate change and psychosocial effects – psychoterrative syndromes)	N/A	N/A	N/A
12.	Alderman, et al. (2012)	Australia	Literature review - systematic review (Flood and human health)	N/A	N/A	N/A

Article #	Article Citation	Country	Literature Type	Study Design (where applicable)	Participants	Outcomes Measured
13.	Alderman et al. (2013)	Australia	Empirical Research Article	Quantitative - Survey (Kessler 6 scale and the PTSD civilian checklist PCL-C 19)	960 flood affected people (during the 2011 Brisbane summer floods) in Australia	Health effects (physical and mental) of 2011 flooding in Brisbane, Australia
14.	Allen et al. (2014).	Global	Literature Review – Narrative review of social factors that influence mental health	N/A	N/A	N/A
15.	Almedom et al. (2008)	Global	Literature Review – Narrative Review on resilience research (policy and practice)	N/A	N/A	N/A
16.	Almedom, et al. (2007)	Eritrea	Empirical Research Article	Quantitative – Survey (Sense of Coherence SOC scale)	265 men and women in Eritrea who have been displaced from rural and urban areas (compared to those who have not been displaced)	Resilience related to displacement
17.	Almedom et al. (2015)	Not specified	Literature Review – theoretical review	N/A	N/A	N/A
18.	Ampuero et al., (2015)	Chile (Robinson Crusoe Island)	Empirical Research Article	Qualitative - Mental wellbeing impact assessment	Residents of Robinso Curusoe Island	Resilience related to: natural environment, meaningful activities,

Article #	Article Citation	Country	Literature Type	Study Design (where applicable)	Participants	Outcomes Measured
				(post disaster) via interviews and fieldwork observations		food, social activities, education, transport and security.
19.	Anderson (2009)	Australia	Empirical Research Article	Qualitative – ethnographic case study via oral histories	Rural dwellers in dryland farm communities of the semiarid Victorian Mallee	Lived experience of coping with climate change related-drought
20.	Anderson et al. (2011)	Global	Literature Review – Narrative Review of mental health and wellbeing	N/A	N/A	N/A
21.	Anderson et al. (2017)	USA	Literature Review – Climate change and health assessments	N/A	N/A	N/A
22.	Asugeni et al. (2015)	Solomon Islands	Empirical Research Article	Mixed methods research - cross-sectional study (survey including close and open ended questions)	Residents of six low-lying villages in East Malaita, Solomon Islands.<br><br>57 questionnaire respondents	Mental health effect of rising sea levels
23.	Austin et al., (2015)	Canada	Literature Review – Narrative review (public health	N/A	N/A	N/A

Article #	Article Citation	Country	Literature Type	Study Design (where applicable)	Participants	Outcomes Measured
			adaptation to climate change)			
24.	Azuma et al. (2014)	Japan	Empirical Research Article	Quantitative – Survey	Flooded residents (379 respondents) in six areas of Japan.	Health status (physical and mental) of residents with water-damaged homes post-flood
25.	Bajayo (2012)	Australia	Literature Review – Narrative Review (community resilience to climate change via public health planning)	N/A	N/A	N/A
26.	Bardsley et al. (2012)	Australia	Literature Review – Narrative Review (climate change vulnerability for the Alinytjara Wilurara Natural Resources Management region)	N/A	N/A	N/A
27.	Barnett et al. (2015)	Australia	Literature Review – Narrative Review (climate change adaptation)	N/A	N/A	N/A

Article #	Article Citation	Country	Literature Type	Study Design (where applicable)	Participants	Outcomes Measured
28.	Bei et al. (2013)	Australia	Empirical Research Article	Quantitate – Survey (surveying for anxiety, PTSD, self-report health, satisfaction with life)	274 older adults (age 60+) before and after flood exposure	Health outcomes (physical and mental) of older adults
29.	Bélanger et al. (2014)	Canada	Empirical Research Article	Mixed Methods (Survey and interviews - cross-sectional household survey)	Residents of low income neighbourhoods (in 9 cities in Canada)	Adverse health effects of heat
30.	Bell (2012)	USA	Literature Review – Narrative Review (whole-systems approach for climate change and health policies)	N/A	N/A	N/A
31.	Bergström et al. 2014	Not specified	Literature Review – Theoretical review (macro, micro, meso understanding of resilience – nature of resilience)	N/A	N/A	N/A
32.	Berkes et al. (2013)	Not specified	Literature Review – Narrative Review (on community resilience from a	N/A	N/A	N/A

Article #	Article Citation	Country	Literature Type	Study Design (where applicable)	Participants	Outcomes Measured
			social-ecological systems perspective and from the psychology of development and mental health)			
33.	Berry et al. (2008a)	Australia	Empirical Research Article	Quantitative – Analysis of statistic census geographic system	Residents of rural Australia	Mental health effects of climate change
34.	Berry et al. (2008b)	Australia	Literature Review – Narrative Review (Rural mental health implications of climate change)	N/A	N/A	N/A
35.	Berry et al. (2009a)	Australia	Empirical Research Article	Quantitative - Survey (self-report on social capital and psychological distress)	963 Residents of Eurobella Shire in New South Wales, Australia (aged 19-97)	Social capital's relationship to mental health
36.	Berry et al. (2009b)	Global	Literature Review – Narrative Review (Climate change impacts to mental health and opportunities for public health)	N/A	N/A	N/A

Article #	Article Citation	Country	Literature Type	Study Design (where applicable)	Participants	Outcomes Measured
37.	Berry et al., (2010a)	Global	Literature Review – Narrative Review (mental health risks and impacts from climate change)	N/A	N/A	N/A
38.	Berry et al., (2010b)	Australia	Literature Review – Narrative Review (climate change and mental health adaptation)	N/A	N/A	N/A
39.	Berry et al. (2011)	Australia	Literature Review – Review of epidemiological research on Farmer's mental health risks related to climate change	N/A	N/A	N/A
40.	Birkmann et al. (2010)	Sri Lanka and Indonesia	Literature Review – Narrative Review (Response related to climate related extreme events and disasters)	N/A	N/A	N/A
41.	Bonanno (2012)	Not Specified	Literature Review – Narrative Review (Resilience constructs related	N/A	N/A	N/A

Article #	Article Citation	Country	Literature Type	Study Design (where applicable)	Participants	Outcomes Measured
			to psychosocial health)			
42.	Bourque et al. (2014)	Canada	Literature Review – Narrative Review (Climate change as an opportunity for public mental health)	N/A	N/A	N/A
43.	Bowles (2015)	Australia	Literature Review – Narrative Review (Climate change and health adaptation (physical and mental) amongst indigenous	N/A	N/A	N/A
44.	Bragg et al. (2012)	Australia and New Zealand	Literature Review – Narrative Review (Ecopsychology and environmental/ecological change)	N/A	N/A	N/A
45.	Brown (2015)	Global	Grey Literature (Resilience, development and global change)	N/A	N/A	N/A
46.	Brown et al. (2011)	Not specified	Literature Review – Narrative	N/A	N/A	N/A

Article #	Article Citation	Country	Literature Type	Study Design (where applicable)	Participants	Outcomes Measured
			Review (resilience, agency, capacity related to environmental change)			
47.	Bunch (2011)	Not specified	Literature Review – Narrative Review (social-ecological resilience, health and well-being)	N/A	N/A	N/A
48.	Bunyavanich et al. (2003)	Not specified	Literature Review – Narrative Review (climate change impacts on child health)	N/A	N/A	N/A
49.	Burch et al. (2014)	Canada,	Literature Review – Narrative Review (Transformational change related to climate change responses)	N/A	N/A	N/A
50.	Burch et al. (2010)	Canada, BC	Empirical Research Article	Qualitative - Case Studies in three municipalities in British Columbia (BC)	Three BC municipalities	Municipal action on climate change (adaptation and mitigation)

Article #	Article Citation	Country	Literature Type	Study Design (where applicable)	Participants	Outcomes Measured
51.	Burton et al. (2016)	Canada	Literature Review – Narrative Review (Psychosocial and physical health consequences of flooding)	N/A	N/A	N/A
52.	Butler et al, (2014)	Global	Literature Review – Narrative Review (climate change impacts on mental health and cognition)	N/A	N/A	N/A
53.	Berkes, (2001)	Canada	Empirical Research Article	Qualitative – Case Study of the adaptive capacity to climate change of residents of Sachs Harbour	Residents of Sachs Harbour	Adaptive capacity to climate change
54.	Cameron (2012)	Canada	Literature Review – Narrative Review (Critique of vulnerability and adaptation approaches (related to climate change) for Indigenous peoples in Canada's arctic	N/A	N/A	N/A

Article #	Article Citation	Country	Literature Type	Study Design (where applicable)	Participants	Outcomes Measured
55.	Cardwell et al. (2013)	Canada	Empirical Research Article	Qualitative – Semi-structured interviews about perceptions of climate change and health	22 residents of Golden Horseshoe (Southern Ontario)	Perceptions of climate change impacts to human health
56.	Carnie et al. (2011)	Australia	Empirical Research Article	Qualitative - Interviews in school-based forums about drought-related mental health experiences	Youth and teachers in rural NSW, Australia	Mental health outcomes related to drought
57.	Carroll et al. (2010)	England	Empirical Research Article	Qualitative – Interviews and focus groups with people who had been flooded and agency workers	Flooded residents of Carlisle northwest England and agency workers working with flooded residents.	Health (physical and mental) and social impacts of a flood disaster
58.	Carroll et al. (2009)	England	Empirical Research Article	Qualitative – phenomenological study	Flooded residents of Carlisle northwest England and agency workers working with flooded residents.	Health (physical and mental) and social impacts of a flood disaster
59.	Castleden et al. (2011)	Not specified	Literature Review – Systematic Review with a narrative summary on concepts of	N/A	N/A	N/A

Article #	Article Citation	Country	Literature Type	Study Design (where applicable)	Participants	Outcomes Measured
			resilience related to public health			
60.	Chand et al. (2008)	Global	Literature Review – Narrative Review of climate change impacts to mental health	N/A	N/A	N/A
61.	Cheng, et al. (2013)	Canada	Literature Review – Narrative review of the health co-benefits and risks of adaptation strategies to climate change	N/A	N/A	N/A
62.	Cheshire et al. (2015)	Not Specified	Literature Review – Narrative review (of community resilience, social capital and governance related to global change)	N/A	N/A	N/A
63.	Chowdhury et al. (2011)	Canada	Literature Review – Narrative Review (of climate change and psychosocial impacts)	N/A	N/A	N/A
64.	Cameron et al. (2015)	Canada Nunavut	Literature Review – Narrative Review (of climate	N/A	N/A	N/A

Article #	Article Citation	Country	Literature Type	Study Design (where applicable)	Participants	Outcomes Measured
			change adaptation and resilience in Nunavut, Canada)			
65.	Clarke et al. (2012)	Canada	Empirical Research Article	Qualitative -Case study (interviews and case study of climate change and health adaptation frameworks in Ontario)	Health Officials	Utility of climate change adaptation frameworks to address health impacts.
66.	Clayton et al. (2014)	USA	Literature Review – Narrative Review of climate change impacts to mental health	N/A	N/A	N/A
67.	Clayton et al. (2017)	USA	Literature Review – Narrative Review of climate change impacts to mental health	N/A	N/A	N/A
68.	Congues, (2014).	Australia	Literature Review – Narrative Review of well-being related to Drought in Australia	N/A	N/A	N/A
69.	Coombe et al. (2015)	Australia	Literature Review - Systematic review (of teacher-mediated	N/A	N/A	N/A

Article #	Article Citation	Country	Literature Type	Study Design (where applicable)	Participants	Outcomes Measured
			interventions to support child mental health post disaster)			
70.	Costello et al. (2009)	Global	Literature Review – Narrative Review (of managing the health effects of climate change)	N/A	N/A	N/A
71.	Cox et al. (2014)	Canada	Empirical Research Article	Qualitative – field testing of rural resilience index (including semi-structured interviews and focus groups)	Rural, remote coastal communities in Canada	Assessment of disaster resilience
72.	Crabtree (2012)	Developing Countries	Literature Review – Narrative Review (of epidemiological literature related to the mental health implications of climate change-related flooding in developing countries.	N/A	N/A	N/A
73.	Crabtree. (2013)	Developing Countries	Literature Review – Narrative	N/A	N/A	N/A

Article #	Article Citation	Country	Literature Type	Study Design (where applicable)	Participants	Outcomes Measured
			Review (of psychosocial resilience post-flood)			
74.	Cunsolo Willox et al. (2012)	Canada	Empirical Research Article	Qualitative - Case study of Nunatsiavut (Interview and descriptive analysis of questionnaire)	Residents of Nunatsiavut (72 in-depth interviews, and 112 descriptive questionnaires)	Climate change impacts on a sense of place
75.	Cunsolo Willox et al. (2013a)	Canada	Empirical Research Article	Qualitative – Case study of Nunatsiavut (interviews)	Residents of Nunatsiavut	Climate change impacts on health and wellbeing
76.	Cunsolo Willox et al. (2013b)	Canada	Empirical Research Article	Qualitative – Case study of Nunatsiavut (67 in-depth interviews)	Community members and local and regional health professionals of Nunatsiavut	Climate change impacts on mental health
77.	Cunsolo Willox et al. (2014)	Canada	Empirical Research Article	Qualitative – Case study of Nunatsiavut	Community members and local and regional health professionals of Nunatsiavut	Climate change impacts on mental health
78.	Cusack et al. (2011)	Not specified	Literature Review – Narrative review (of the impact of heatwaves on people with	N/A	N/A	N/A

Article #	Article Citation	Country	Literature Type	Study Design (where applicable)	Participants	Outcomes Measured
			substance abuse and mental health conditions)			
79.	Cutter et al. (2008)	USA	Grey Literature (of research on community resilience related to hazards)	N/A	N/A	N/A
80.	Davidson et al. (2006)	Global	Literature Review – Narrative Review (of risks and impacts of disaster, lack of access to care impacts abilities to adapt to disasters)	N/A	N/A	N/A
81.	Dean et al. (2010)	Australia	Empirical Research Article	Mixed methods research (focus groups and self-report questionnaire; strengths and difficulties questionnaire and a drought and community survey for children comprised the self-report survey)	Male and female (n =111) adolescents in he Riverina region of New South Wales.	Adolescent mental health experiences of drought

Article #	Article Citation	Country	Literature Type	Study Design (where applicable)	Participants	Outcomes Measured
82.	Dean et al. (2011)	Not specified	Literature Review – Narrative review (of the relationship between biodiversity and improved mental health in urban environments)	N/A	N/A	N/A
83.	Deeming et al., (2014)	Europe	Literature Review – Narrative review (of resilience and adaptation related to hydrometeorological hazards based on 5 case studies across Europe)	N/A	N/A	N/A
84.	Deppisch et al. (2013)	Not specified	Literature Review – Narrative review (of applying social-ecological resilience thinking to transdisciplinary research on climate change adaptation)	N/A	N/A	N/A
85.	Dodgen et al. (2016)	USA	Literature Review – Narrative review (of the mental health implications	N/A	N/A	N/A

Article #	Article Citation	Country	Literature Type	Study Design (where applicable)	Participants	Outcomes Measured
			of climate change in the USA)			
86.	Doherty et al. (2011)	Global	Literature Review – Narrative review (of the psychological impacts of climate change)	N/A	N/A	N/A
87.	Dorji (2006)	Bhutan	Literature Review – Narrative review (of the psychosocial and mental health implications of disaster in Bhutan)	N/A	N/A	N/A
88.	Douglas et al. (2012)	USA	Empirical Research Article	Qualitative – Case study of two metropolitan Boston, Mass. Communities (workshops with residents)	Residents of two metropolitan cities in Boston, Mass.	Perceived adaptation to climate change
89.	Durkalec et al. (2015)	Canada	Empirical Research Article	Qualitative -Case study of Inuit community in Nain (participant observation, focus groups, and interviews)	Nain residents; interviews (n= 22), focus groups (n=2), and participant observation	The impact of climate change on a sense of place for Inuit community

Article #	Article Citation	Country	Literature Type	Study Design (where applicable)	Participants	Outcomes Measured
90.	Ebi et al. (2016)	Not specified	Literature review – Narrative review (of health vulnerabilities related to extreme events)	N/A	N/A	N/A
91.	Edwards et al., (2015)	Australia	Empirical Research Article	Quantitative – Survey (rural and regional areas of Australia)	8,000 residents of rural and regional Australia	Impact of drought on mental health
92.	Edwards et al. (2011).	Not specified	Grey Literature (of climate change, resilience and transformation related to climate change and human health and wellbeing)	N/A	N/A	N/A
93.	Eichler (2015)	Canada	Literature Review – Narrative review (of climate change coping for children)	N/A	N/A	N/A
94.	Eisenman et al. (2015)	USA	Empirical Research Article	Quantitative – household survey (Kessler Distress Scale)	Residents of Wallow Fire Arizona (n= 1387 households)	Psychological distress related to wildfire
95.	Evans et al. (2016)	Australia	Empirical Research Article	Qualitative - Key informant interviews and	Representatives of the fisheries and tourism sectors of the	Psychosocial and structural limits to

Article #	Article Citation	Country	Literature Type	Study Design (where applicable)	Participants	Outcomes Measured
				multi-stakeholder workshops	Great Barrier Reef region	climate change adaptation
96.	Every-Palmer et al. (2016)	Australia	Literature Review – Narrative review (of the role for psychiatry in the field of climate change)	N/A	N/A	N/A
97.	Fernandez et al. (2015)	Not specified	Literature Review – Systematic mapping review (of flood impacts to mental health in river catchments)	N/A	N/A	N/A
98.	Few et al. (2004)	Not specified	Literature Review: Narrative review (of climate related-floods and health)	N/A	N/A	N/A
99.	Fleming et al. (2015)	Australia	Empirical Research Article	Quantitative – Survey (perceptions of how climate change affects wine industry)	50 Wine Industry Growers	Stress related to climate change impacts to wine industry growers
100.	Ford et al. (2012)	Canada	Literature Review – Narrative Review (of Indigenous health related to climate change)	N/A	N/A	N/A

Article #	Article Citation	Country	Literature Type	Study Design (where applicable)	Participants	Outcomes Measured
101.	Ford et al. (2016)	Canada	Empirical Research Article	Qualitative – Workshops on community-based adaptation approaches to research	Sample of Inuit residents in Canadian Arctic	Role of community-based adaptation research in climate change research
102.	Fresque-Baxter et al. (2012)	Not specified	Literature Review – Narrative review (of place and identify related to climate change adaptation)	N/A	N/A	N/A
103.	Friel et al. (2014)	Australia	Empirical Research Article	Quantitative – Survey (population-based survey)	Sample of Australian adults	Perceived impact of drought on food security and mental health
104.	Fritze et al. (2008)	Global	Literature Review – Narrative review (climate change and mental health)	N/A	N/A	N/A
105.	Fundter et al. (2008)	Netherlands	Literature Review – Narrative review (of health impacts related to large-scale floods)	N/A	N/A	N/A
106.	Furgal et al. (2008)	Canada	Literature Review – Narrative review (health impacts of climate change in Canada's north)	N/A	N/A	N/A

Article #	Article Citation	Country	Literature Type	Study Design (where applicable)	Participants	Outcomes Measured
107.	Füssel et al. (2007)	Not specified	Literature Review – Narrative review (of vulnerability related to climate change research)	N/A	N/A	N/A
108.	Galea et al. (2007)	USA	Empirical Research Article	Quantitative (survey)	Hurricane Katrina survivors	Hurricane-related stressors and mental health
109.	Gibbs et al. (2013)	Australia	Empirical Research Article	Mixed Methods (longitudinal study of 2009 Victorian bushfires in Australia via surveys, interviews, focus groups)	Adults and children (over age 5)	Mental health, wellbeing and social impacts of 2009 wildfires
110.	Gifford (2011)	Not specified	Literature Review – Narrative review (of psychological barriers to climate change mitigation and adaptation)	N/A	N/A	N/A
111.	Goh et al. (2012)	Developing countries	Literature Review – Narrative review (climate change impacts on gender in developing countries)	N/A	N/A	N/A
112.	Gray (2008)	Not Specified	Grey Literature (commentary long-	N/A	N/A	N/A

Article #	Article Citation	Country	Literature Type	Study Design (where applicable)	Participants	Outcomes Measured
			term health impacts of flooding)			
113.	Green et al. (2014)	Australia	Literature Review – Narrative Review (climate change impacts on the health and wellbeing on Indigenous peoples in remote Australian communities)	N/A	N/A	N/A
114.	Green et al. (2012)	Australia	Literature Review – Narrative review (of theory and practice related to climate change vulnerability assessments for remote indigenous communities in Australia)	N/A	N/A	N/A
115.	Greene et al. (2015)	England	Empirical Research Article	Quantitative – Survey (use of Bayesian structure equation modelling to measure latent resilience)	2238 flood affected residents living in Southern in England	Resilience, vulnerability and psychological harm related to flooding

Article #	Article Citation	Country	Literature Type	Study Design (where applicable)	Participants	Outcomes Measured
116.	Greene (2014).	Not Specified	Literature Review – Narrative Review (of resilience and ecological systems)	N/A	N/A	N/A
117.	Gruebner et al. (2015)	USA	Empirical Research Article	Quantitative – Telephone survey (using e Posttraumatic Stress Checklist for DSM-5 (PCL-5) and the nine-item Patient Health Questionnaire (PHQ-9) to measure depression)	418 Adults affected by Hurricane Sandy	PTSD and Depression of people exposed to Hurricane Sandy
118.	Gunn et al. (2012)	Australia	Empirical Research Article	Quantitative – Survey (Kessler Psychological Distress Scale)	309 Drought-affected southern Australian farmers	Stress and coping of farmers related to drought
119.	Gutierrez et al. (2016)	USA	Literature Review – Narrative review (climate justice in rural, southeast, USA)	N/A	N/A	N/A

Article #	Article Citation	Country	Literature Type	Study Design (where applicable)	Participants	Outcomes Measured
120.	Hanigan et al. (2012)	Australia	Empirical Research Article	Quantitative – Review of data between 1907-2007 on suicides (using generalized additive model that controlled for season, region, and long-term suicide trends)	Suicide data in Australia from 1907-2007	Suicides related to drought
121.	Hannigan et al. (2011)	UK	Literature Review – Theoretical Review (of policies related to mental health in the UK)	N/A	N/A	N/A
122.	Harper et al. (2015)	Canada	Empirical Research Article	Mixed Methods – in-depth interviews, and photovoice workshops	Regional health representatives in Nunatsiavut (11 interviews; 3 photovoice workshops (with 11 Rigolet community members)	Climate-sensitive health priorities.
123.	Hart et al. (2011)	Australia	Literature Review – Narrative Review (of drought related	N/A	N/A	N/A

Article #	Article Citation	Country	Literature Type	Study Design (where applicable)	Participants	Outcomes Measured
			mental health in New South Wales)			
124.	Henderson et al. (2015)	Not Specified	Literature Review – Narrative Review (climate change and mental disorders)	N/A	N/A	N/A
125.	Höfler (2014).	Not Specified	Literature Review – Narrative Review (of psychosocial resilience in adults related to disasters)	N/A	N/A	N/A
126.	Holmes (2015)	Trinidad and Tobago	Literature Review – Narrative Review (of risk and resilience related to climate hazards and extreme weather)	N/A	N/A	N/A
127.	Holmgren (2012)	Not Specified	Grey Literature (of adapting to climate change and peak oil)	N/A	N/A	N/A
128.	Hunter (2009).	Australia	Literature Review – Narrative Review (of effects of climate change on mental health of	N/A	N/A	N/A

Article #	Article Citation	Country	Literature Type	Study Design (where applicable)	Participants	Outcomes Measured
			Indigenous peoples in northern Australia)			
129.	Hutton (2005)	Canada	Literature Review – Narrative Review (Psychosocial consequences of climate change)	N/A	N/A	N/A
130.	Imperiale et al. (2016)	Italy	Literature Review – Narrative Review (community resilience post-disaster in L'Aquila)	N/A	N/A	N/A
131.	Inder et al. (2011)	Australia	Empirical Research Article	Quantitative – Cohort case study using surveys (Household, Income and Labour Dynamics in Australia Survey; Australian Rural Mental Health Study; Hunter Community Study; and Extending Treatments,	Rural and remote population in Australia	Climate related mental health adversities

Article #	Article Citation	Country	Literature Type	Study Design (where applicable)	Participants	Outcomes Measured
				Education and Networks in Depression study)		
132.	Jain, (2015)	Global	Other – Commentary addressing questions about doing mental health justice in the SDG)	N/A	N/A	N/A
133.	Jane-Llopis et al. (2011)	Not Specified	Literature Review – Narrative Review (addressing the burden of mental health)	N/A	N/A	N/A
134.	Jaspal et al. (2014)	Not Specified	Literature Review – Theoretical Review (Using social representation theory and identity process theory trying to understand human responses to climate change)	N/A	N/A	N/A
135.	Jenkins et al. (2011)	Global	Literature Review – Narrative	N/A	N/A	N/A

Article #	Article Citation	Country	Literature Type	Study Design (where applicable)	Participants	Outcomes Measured
			Review (mental health and global development)			
136.	Joerin et al, (2014)	Global	Literature Review -Theoretical Review (concept of resilience related to disasters)	N/A	N/A	N/A
137.	Jones et al. (2012)	Australia	Empirical Research Article	Mixed Methods Research – interview and self-report survey of behaviours	50 Patients with OCD	OCD related to climate change
138.	Keim (2008)	Global	Literature Review – Narrative review (of public health's role in climate change adaptation)	N/A	N/A	N/A
139.	Keim (2011)	Global	Literature Review – Narrative Review (public health's role in reducing vulnerability to climate change)	N/A	N/A	N/A
140.	Keller (2013)	France	Literature Review – Narrative Revie (Paris Heatwave)	N/A	N/A	N/A

Article #	Article Citation	Country	Literature Type	Study Design (where applicable)	Participants	Outcomes Measured
141.	Kessler (2008)	USA	Empirical Research Article	Quantitative – Surveys (TSQ for mood and PTSD, and Kessler 6)	815 residents who experienced Hurricane Katrina	Mental illness and suicidality
142.	Kirmayer et al. (2011)	Canada	Literature Review – Narrative Review (of resilience constructs from the perspectives of indigenous peoples - Inuit, Métis, Mi'kmaq, and Mohawk)	N/A	N/A	N/A
143.	Kjellstrom et al. (2013)	Global	Literature Review – Narrative Review (of non-communicable health risks of climate change to health and wellbeing)	N/A	N/A	N/A
144.	Koger et al. (2011)	Not Specified	Literature Review – Narrative Review (psychological solutions and strategies to address climate change)	N/A	N/A	N/A

Article #	Article Citation	Country	Literature Type	Study Design (where applicable)	Participants	Outcomes Measured
145.	Kuehne (2014)	Australia	Empirical Research Article	Qualitative – Interviews	Key informants irrigators from the South Australian Riverland (n = 11)	Farmers perceptions of climate change and adaptation responses
146.	Kukarenko, (2011)	Arctic Region	Literature Review – Narrative Review (of human health, gender, and climate change in the Arctic)	N/A	N/A	N/A
147.	LaLone (2012)	USA	Empirical Research Article	Qualitative - Ethnographic case study of rural Appalachia	Sample of people who experienced tornados in rural Appalachia	Social capital mobilization post-disaster
148.	Lamond (2014)	Not Specified	Literature Review – Narrative Review (of impact of repeat flooding on mental health)	N/A	N/A	N/A
149.	Lang (2015)	Not Specified	Grey Literature (of climate change and mental health)	N/A	N/A	N/A
150.	Leff (2008)	Not Specified	Literature Review – Narrative Review (climate change and psychiatry)	N/A	N/A	N/A

Article #	Article Citation	Country	Literature Type	Study Design (where applicable)	Participants	Outcomes Measured
151.	Leslie et al (2013)	East Africa	Empirical Research Article	Qualitative Study – Case Study	Sample of pastoral East African Population	Responses to environmental change
152.	Levine et al. (2011)	Africa	Literature Review – Narrative Review (adaptive capacity and climate change in Africa)	N/A	N/A	N/A
153.	Levy et al. (2015)	Global	Literature Review – Narrative Review (climate change social justice and human rights)	N/A	N/A	N/A
154.	Lindahl et al. (2013)	Not Specified	Grey Literature (mental health, health equity, and social sustainability)	N/A	N/A	N/A
155.	Loboda (2014)	Arctic	Literature Review – Narrative Review (adaptation and climate change in the Arctic)	N/A	N/A	N/A
156.	Lorenz et al. (2016)	Not Specified	Literature Review – Narrative Review (of	N/A	N/A	N/A

Article #	Article Citation	Country	Literature Type	Study Design (where applicable)	Participants	Outcomes Measured
			resilience in disasters)			
157.	Lowe et al. (2013)	OECD Member countries	Literature Review – Systematic Review (of health vulnerabilities – related to floods)	N/A	N/A	N/A
158.	Luber et al. (2015)	Global	Grey Literature Review (climate change and human health)	N/A	N/A	N/A
159.	MacDonald et al. (2015)	Canada	Empirical Research Article	Qualitative – In-depth interviews	Youth aged 15–25 from five communities of the Nunatsiavut region of Labrador, Canada	Protective factors that support wellbeing in a changing climate
160.	Manyena (2014)	Zimbabwe	Empirical Research Article	Qualitative – Case study	Zimbabwe	Discourse and narratives of disaster resilience
161.	Marinucci et al. (2014)	USA	Literature Review – Narrative Review (or resilience in public health to climate change)	N/A	N/A	N/A
162.	McFarlane et al. (2012)	Not Specified	Literature Review – Narrative Review (of mental	N/A	N/A	N/A

Article #	Article Citation	Country	Literature Type	Study Design (where applicable)	Participants	Outcomes Measured
			health services post-disaster)			
163.	Miles et al. (2011)	Not Specified	Literature Review – Theoretical Review (Resilience and disaster modelling)	N/A	N/A	N/A
164.	Molnar (2010)	Global (focus on rural areas)	Literature Review – Narrative Review (of societal responses to climate change in rural areas)	N/A	N/A	N/A
165.	Motesharreiet al. (2016) .	Global	Literature Review – Narrative Review (of sustainability modelling – human and earth systems)	N/A	N/A	N/A
166.	Morello-Frosch et al. (2011)	USA	Empirical Research Article	Qualitative – Observation and interviews	Three community and advocacy-based rebuilding and organizing project	Resilience related to Hurricane Katrina
167.	Morrissey et al. (2007)	Australia	Literature Review – Narrative Review (disaster, climate change, and mental health)	N/A	N/A	N/A

Article #	Article Citation	Country	Literature Type	Study Design (where applicable)	Participants	Outcomes Measured
168.	Morss et al. (2011)	Not Specified	Literature Review – Narrative review (climate change and societal outcomes)	N/A	N/A	N/A
169.	Moser (2013)	Not Specified	Literature Review – Narrative Review (adaptation related to climate change – focus on emotional and political adaptation)	N/A	N/A	N/A
170.	Munro et al. (2017)	England	Empirical Research Article	Quantitative – Survey (Measuring depression PHQ-2; anxiety using Generalised Anxiety Disorder [GAD]-2 anxiety scale, and post-traumatic stress disorder, measured by the Post-Traumatic Stress Disorder Checklist [PCL]-6 scale)	Flooded residents	Mental health outcomes of flooding

Article #	Article Citation	Country	Literature Type	Study Design (where applicable)	Participants	Outcomes Measured
171.	Myers et al. (2011)	Global	Literature Review – Narrative Review (of indirect effects of climate change)	N/A	N/A	N/A
172.	Myers et al. (2012)	Global	Literature Review – Narrative Review (public health, emotions and climate change)	N/A	N/A	N/A
173.	Nahar et al. (2014)	Bangladesh	Literature Review – Narrative Review (mental health care post-disaster in Bangladesh)	N/A	N/A	N/A
174.	Neria et al. (2009)	Not Specified	Grey Literature (Mental health and disasters)	N/A	N/A	N/A
175.	Neria et al. (2012).	USA	Literature Review – Narrative Review (Mental Health effects of Hurricane Sandy)	N/A	N/A	N/A
176.	North et al. (2013)	Global	Literature Review – Narrative review (mental health and community disaster response)	N/A	N/A	N/A

Article #	Article Citation	Country	Literature Type	Study Design (where applicable)	Participants	Outcomes Measured
177.	Nurse et al. (2010)	Global	Literature Review – Narrative Review (climate change and mental health response)	N/A	N/A	N/A
178.	Oba et al. (2010)	Thailand	Empirical Research Article	Qualitative – Focus groups	People who experienced the flood, health professionals, and health volunteers	Perceptions of psychological wellbeing
179.	O'Brien et al. (2010)	Global	Literature Review – Narrative Review (of climate, security, and ethics)	N/A	N/A	N/A
180.	Obrien et al. (2014)	Australia	Empirical Research Article	Quantitative - longitudinal review of rainfall records and survey	Sample of drought-affected rural and urban dwellers	Mental health related to drought
181.	O'Donnell et al. (2016)	Global	Literature Review – Narrative Review (disasters, mental health, older adults)	N/A	N/A	N/A
182.	Ojala (2012)	Sweden	Empirical Research Article	Quantitative – Survey	Swedish Children (n=293)	Coping with climate change
183.	Ojala (2013)	Swedish	Empirical Research Article	Quantitative – Survey	Swedish adolescents (n=321)	Coping with climate change
184.	Ojala (2016)	Not Specified	Literature Review – Narrative review	N/A	N/A	N/A

Article #	Article Citation	Country	Literature Type	Study Design (where applicable)	Participants	Outcomes Measured
			(children, emotions, climate change)			
185.	Opara (2014).	Nigeria	Grey Literature (Climate change in Nigeria)	N/A	N/A	N/A
186.	Ortega-Egea et al. (2014)	Europe	Empirical Research Article	Quantitative - Survey	Sample of European citizens	Psychosocial correlates of climate change mitigation behaviours
187.	Ostry et al. (2010)	Canada	Empirical Research Article	Qualitative – Case study	Communities in BC	Climate Change and health impacts
188.	Padhy et al. (2015)	Not Specified	Literature Review – Narrative Review (of mental health effects of climate change)	N/A	N/A	N/A
189.	Page et al. (2010)	Global	Literature Review – Narrative Review (of climate change and mental health)	N/A	N/A	N/A
190.	Parlee et al. (2012)	Canada	Literature Review – Narrative Review (well-being related to environmental change in the Arctic)	N/A	N/A	N/A

Article #	Article Citation	Country	Literature Type	Study Design (where applicable)	Participants	Outcomes Measured
191.	Parsons et al. (2016)	Global	Literature Review – Narrative Review (disaster resilience)	N/A	N/A	N/A
192.	Petrasek et al (2013)	Canada	Empirical Research Article	Qualitative – Interviews	(12–25 years old) in the Inuit community of Rigolet, Nunatsiavut, Canada (n= 20)	Observations and perceptions of climate change
193.	Phua (2015)	International	Literature Review – Narrative Review (healthcare systems and climate change)	N/A	N/A	N/A
194.	Polain et al. (2011)	Australia	Empirical Research Article	Mixed Methods – Semi-structured content forums and content analysis	One hundred and fifty older farmers, their families, Industry and Investment NSW, rural financial and mental health services, the Country Women's Association and other non-government agencies.	Perceptions of mental health consequences of climate change
195.	Popovski et al. (2012)	Not Specified	Literature Review – Narrative Review (climate change victims)	N/A	N/A	N/A

Article #	Article Citation	Country	Literature Type	Study Design (where applicable)	Participants	Outcomes Measured
196.	Powers et al. (2015)	Australia	Empirical Research Article	Quantitative – Survey (longitudinal, women's health; surveys included the Mental Health Index of the Medical Outcomes Study Short-Form 36)	6,664 women in Australia (between 1996, 1998, 2001, 2004 and 2008) who were born between 1946 and1951	Mental health effects of drought
197.	Rahman et al. (2014)	Global	Literature Review – Narrative Review (of youth perceptions of climate change impacts to health)	N/A	N/A	N/A
198.	Pfefferbaum et al. (2011)	USA	Grey Literature (community resilience to disaster toolkit)	N/A	N/A	N/A
199.	Ramsay et al. (2011)	Global	Grey Literature (PTG, resilience, spirituality related to climate change)	N/A	N/A	N/A
200.	Rataj et al. (2016)	Developing Countries	Literature Review – Systematic Review (extreme weather and mental health disorders)	N/A	N/A	N/A

Article #	Article Citation	Country	Literature Type	Study Design (where applicable)	Participants	Outcomes Measured
201.	Reser et al. (2012)	Not Specified	Literature Review – Narrative Review (coping with climate change)	N/A	N/A	N/A
202.	Reser et al. (2011a)	Not Specified	Literature Review – Narrative Review (psychological responses and adaptation to climate change)	N/A	N/A	N/A
203.	Reser et al. (2011b).	Not Specified	Literature Review – Narrative Review (adaptation and coping with climate change)	N/A	N/A	N/A
204.	Rice et al. (2016)	Asia pacific	Literature Review – Narrative review (climate change and mental health)	N/A	N/A	N/A
205.	Riemer et al. (2011)	Global	Literature Review – Narrative Review (climate change and psychology)	N/A	N/A	N/A
206.	Rigby et al. (2011).	Australia	Empirical Research Article	Mixed Methods Research (content	Aboriginal people, service providers and other stakeholders	Perceived impact of drought on social and emotional wellbeing

Article #	Article Citation	Country	Literature Type	Study Design (where applicable)	Participants	Outcomes Measured
				analysis and semi-structured forums)		
207.	Rodriguez-Llanes et al. (2013)	Not Specified	Literature Review – Narrative Review (measuring psychological resilience to disasters)	N/A	N/A	N/A
208.	Roeser (2012)	Not Specified	Literature Review – Narrative Review (communication and engagement related to climate change and emotions)	N/A	N/A	N/A
209.	Roufeil et al. (2014)	Australia	Literature Review – Narrative Review (health challenges in rural Australian communities)	N/A	N/A	N/A
210.	Rudolph, et al. (2015)	Global	Literature Review (Narrative Review – climate change and health inequities)	N/A	N/A	N/A
211.	Rufat et al. (2015)	Global	Literature Review (Narrative Review	N/A	N/A	N/A

Article #	Article Citation	Country	Literature Type	Study Design (where applicable)	Participants	Outcomes Measured
			of case studies– social vulnerability and floods)			
212.	Sahni et al. (2016)	Canada	Empirical Research Article	Quantitative – Public health surveillance (emergency department visits, administrative data)	People who experienced the 2013 flood	Health events (including mental health)
213.	Saniotis et al. (2010)	Australia	Literature Review – Narrative Review (climate change impacts on older adults)	N/A	N/A	N/A
214.	Sapiains et al. (2016)	Not Specified	Empirical Research Article	Qualitative	People who thought climate change is natural and those who thought it is human-induced (N=156)	Individual responses to climate change
215.	Satcher et al. (2007)	USA	Literature Review – Narrative Review (of disasters and mental health)	N/A	N/A	N/A
216.	Schmeltz et al. (2013)	USA	Empirical Research Article	Qualitative -field report from Hurricane Sandy	Street canvasses, governmental reports, community flyers, and meeting	Accounts of the effects of Sandy and the response to daily needs

Article #	Article Citation	Country	Literature Type	Study Design (where applicable)	Participants	Outcomes Measured
					transcripts, as well as firsthand observations by a local nonprofit Red Hook Initiative (RHI) and community members, and social media	
217.	Schrader et al (2013)	Not Specified	Other – commentary (mental health researchers learning from climate change debate)	N/A	N/A	N/A
218.	Schulte et al. (2016)	Not Specified	Literature Review – Narrative Review (climate change and occupational health)	N/A	N/A	N/A
219.	Schulte et al. (2009)	Not Specified	Literature Review – Narrative Review and framework (climate change and occupational health)	N/A	N/A	N/A
220.	Shultz et al. (2017)	USA	Other – Commentary	N/A	N/A	N/A

Article #	Article Citation	Country	Literature Type	Study Design (where applicable)	Participants	Outcomes Measured
			(health consequences of Hurricane Karvey)			
221.	Searle et al. (2009)	Not Specified	Literature Review – Narrative Review (psychological impacts of climate change)	N/A	N/A	N/A
222.	Searle et al. (2010)	Australia	Empirical Research Article	Quantitative – Survey (Cross-sectional)	Sample of adults in Australia (N= 275)	Perceptions of distress related to climate change
223.	Selvey et al. (2015)	Not Specified	Literature Review – Narrative Review (health and climate change)	N/A	N/A	N/A
224.	Shaw et al. (2009)	Canada	Empirical Research Article	Qualitative – Participatory capacity building approach	Community of Delta	Climate change scenarios for capacity building
225.	Shaw et al. (2014)	UK	Empirical Research Article	Mixed Methods – Case study (interview, discourse review)	Residents of flood prone area; health service providers (interviews), community facilities (discourse review)	Social vulnerability and social resilience
226.	Sharifi et al. (2016)	Not Specified	Literature Review – Narrative	N/A	N/A	N/A

Article #	Article Citation	Country	Literature Type	Study Design (where applicable)	Participants	Outcomes Measured
			Review (of community resilience assessment tools)			
227.	Shukla. (2013)	Global	Literature Review – Narrative Review (extreme weather and mental health)	N/A	N/A	N/A
228.	Silove et al. (2006)	Not Specified	Literature Review – Narrative Review (community psychosocial needs post-disaster)	N/A	N/A	N/A
229.	Smoyer-Tomic et al. (2004)	Canada	Literature Review – Narrative Review (health consequences of drought in Canadian prairies)	N/A	N/A	N/A
230.	Simpson et al. (2011)	Global	Literature Review – Narrative Review (mental health, wellbeing, climate change)	N/A	N/A	N/A
231.	Spence et al. (2012)	USA	Empirical Research Article	Quantitative - Survey	Sample of American public (n=653)	Perceptions about the psychological implications of climate change

Article #	Article Citation	Country	Literature Type	Study Design (where applicable)	Participants	Outcomes Measured
232.	Stain et al. (2011)	Australia	Empirical Research Article	Quantitative – Survey (Kessler 10 distress scale)	Adults in rural areas exposed to chronic drought (n= 302)	Psychological impact of chronic drought
233.	Stanke et al. (2012)	UK	Literature Review – Narrative Review (mental health consequences of prolonged drought)	N/A	N/A	N/A
234.	Stedman (2004)	Canada	Empirical Research Article	Quantitative - Survey	Policy actors in Canada (n=851)	Perceptions of climate change risks
235.	Stephenson et al. (2014)	Not Specified	Literature Review – Narrative Review (long-term health impacts of flood)	N/A	N/A	N/A
236.	Stuart et al. (2011)	New Zealand	Literature Review – Narrative Review (of health education and response to vulnerable people during heat waves)	N/A	N/A	N/A
237.	Sung et al. (2011)	Taiwan	Empirical Research Article	Quantitative - In patient data interpolated with meteorological data (longitudinal study)	Psychiatric Inpatient Medical Claim dataset of the National Health Insurance Research Database.	Schizophrenia related to daily temperatures

Article #	Article Citation	Country	Literature Type	Study Design (where applicable)	Participants	Outcomes Measured
238.	Swim et al. (2009)	USA	Literature Review- Narrative Review (psychology and climate change)	N/A	N/A	N/A
239.	Swim et al. (2011)	USA	Literature Review- Narrative Review (Human behaviour, psychology and climate change)	N/A	N/A	N/A
240.	Swim et al (2011	USA	Literature Review – Narrative Review (Psychology and climate change)	N/A	N/A	N/A
241.	Syal et al. (2011)	USA	Empirical Research Article	Quantitative – Survey	U.S. Environmental health directors	Individual level beliefs about climate change and human health
242.	Tapsell (2010)	Not Specified	Literature Review – Narrative Review (of psychological impacts of flooding)	N/A	N/A	N/A
243.	Thomas et al. (2014)	Not Specified	Literature Review – Narrative Review (climate change impacts on	N/A	N/A	N/A

Article #	Article Citation	Country	Literature Type	Study Design (where applicable)	Participants	Outcomes Measured
			health and wellbeing)			
244.	Thornley et al. (2015)	New Zealand	Empirical Research Article	Qualitative – focus groups and interviews	92 community-based leaders and residents participated in 11 focus groups and 29 interviews	Community response to earthquake
245.	Tierney, K. (2014)	Global	Grey Literature (Disasters and social and institutional factors)	N/A	N/A	N/A
246.	THRIVE (2004)	Various	Grey Literature (community resilience to health disparities - toolkit)	N/A	N/A	N/A
247.	Tobias et al. (2014).	Canada	Empirical Research Article	Qualitative – Community based research (Anishinaabe communities in Ontario, Canada; in-depth interviews	Elders of Anishinaabe community (n=46)	Environmental impacts of land dispossession
248.	Tong et al. (2010)	Not Specified	Literature Review – Narrative Review (environmental	N/A	N/A	N/A

Article #	Article Citation	Country	Literature Type	Study Design (where applicable)	Participants	Outcomes Measured
			health and climate change)			
249.	Tonna et al. (2009)	Australia	Literature Review - Narrative Review (mental health and drought)	N/A	N/A	N/A
250.	Tosone et al. (2015)	USA	Empirical Research Article	Quantitative - Survey	244 social workers from New Orleans	Shared Traumatic Stress
251.	Trombley et al. (2017)	USA	Literature Review – Narrative Review (climate change and mental health)	N/A	N/A	N/A
252.	Trang et al. (2016)	Vietnam	Empirical Research Article	Quantitative - Hospital admission data	Database from Hanoi Mental Hospital covering 5 years from 2008 to 2012 was used	Mental health disorders
253.	Truelove et al. (2015)	Sri Lanka	Empirical Research Article	Qualitative – Case study	192 paddy farmers from five villages in the dry zone of Sri Lanka.	Perceptions of drought risk perceptions, beliefs about efficacy, village identification, and perceived descriptive norms
254.	Turner et al. (2012).	Australia	Empirical Research Article	Quantitative - Survey	2011 floods on residents of the	Health impacts of the 2011 flood (mental and physical)

Article #	Article Citation	Country	Literature Type	Study Design (where applicable)	Participants	Outcomes Measured
					greater Brisbane region	
255.	Tunstall et al. (2006).	England and Wales	Empirical Research Article	Mixed Methods Research – Survey (General Health Questionnaire and interview)	Sample of residents in 30 flood-affected areas	Health effects of flooding
256.	Van Kessel et al. (2014)	Not Specified	Literature Review – Systematic Review (resilience and disaster interventions)	N/A	N/A	N/A
257.	Vasseur et al. (2015)	Canada	Empirical Research Article	Qualitative - Interviews	Men and women from 10 coastal communities in three provinces (Quebec, New Brunswick, and Prince Edward Island)	Perceptions and reactions to extreme weather
258.	Verplanken et al. (2013)	US and Europe	Empirical Research Article	Quantitative – Survey	132 university men and women	Ecological worrying, environmental attitudes, prevalence of pro-environmental behaviors, pathological worrying, and the Big Five personality traits.

Article #	Article Citation	Country	Literature Type	Study Design (where applicable)	Participants	Outcomes Measured
259.	Vida et al. (2012).	Canada	Empirical Research Article	Quantitative – emergency department visits related to three geographic areas experiencing increased ambient temperature and humidity	Under age 65 and over age 65 for three geographic areas in Quebec	Mood and behavioural disorders
260.	Vins et al. (2015)	Not Specified	Literature Review – Systematic Review (mental health outcomes related to drought)	N/A	N/A	N/A
261.	Wainwright et al. (2009)	Global	Literature Review – Narrative Review (climate change and psychology)	N/A	N/A	N/A
262.	Waite et al. (2017)	England	Empirical Research Article	Quantitative – Survey (cross-sectional; survey instruments included: Patient Health Questionnaire, Generalized Anxiety Disorder, and Post	Households that had been flooded between Dec. 2013 and March 2014	Mental health outcomes of flood

Article #	Article Citation	Country	Literature Type	Study Design (where applicable)	Participants	Outcomes Measured
				Traumatic Stress Checklist)		
263.	Wang et al. (2014).	Canada	Empirical Research Article	Quantitative – Emergency department visits related to ambient temperatures	People who visited Toronto Emergency Room for behavioural and mental disorders during extreme temperatures.	Mental and behavioural disorders
264.	Weissbecker (2011)	Global	Grey Literature (Climate change and human well-being)	N/A	N/A	N/A
265.	Wenden (2011).	Global	Grey Literature (Climate change, vulnerability, and women)	N/A	N/A	N/A
266.	West et al. (2008)	USA	Empirical Research Article	Quantitative – Cross-sectional survey	New Orleans Police Department (NOPD) personnel who provided law enforcement and relief services to affected communities following Hurricane Katrina.	Post-Traumatic Stress Disorder
267.	White et al. (2016)	Global	Literature Review – Narrative Review (global	N/A	N/A	N/A

Article #	Article Citation	Country	Literature Type	Study Design (where applicable)	Participants	Outcomes Measured
			mental health and wellbeing)			
268.	Wilby et al. (2012)	Various	Literature Review – Narrative Review (climate change adaptation to floods)	N/A	N/A	N/A
269.	Wilson et al. (2010)	USA	Empirical Research Article	Quantitative - GIS mapping	Areas vulnerable to climate change in the USA	Social and environmental determinants
270.	Winkler et al. (2015)	Not Specified	Literature Review – Narrative Review (of mitigation and adaptation related to climate change)	N/A	N/A	N/A
271.	Wolf et al. (2015)	Canada	Empirical Research Article	Qualitative- Q interview methods	Sample of people in two Labrador communities	Values and traditional practices in climate change adaptation
272.	Wolkin et al. (2015)	USA	Empirical Research Article	Qualitative – Interviews and workshops	Nine key informant interviews were conducted with emergency managers; workshop with emergency managers	Social vulnerabilities and approaches to disaster management
273.	Wulff et al. (2015)	Not Specified	Literature Review – Narrative Review (health	N/A	N/A	N/A

Article #	Article Citation	Country	Literature Type	Study Design (where applicable)	Participants	Outcomes Measured
			resilience to disasters)			
274.	Yusa et al. (2015)	Canada	Literature Review – Narrative Review (climate change, drought, human health)	N/A	N/A	N/A
275.	Zakour et al. (2013)	Not Specified	Grey Literature (disaster vulnerability and community)	N/A	N/A	N/A
276.	Zaremohzzabieh et al. (2013)	Not Specified	Literature Review – Narrative Review (Role of Internet in flood protection and wellbeing for youth)	N/A	N/A	N/A

References

1. Adger, W. N., Barnett, J., Chapin Iii, F. S., & Ellemor, H. (2011). This must be the place: underrepresentation of identity and meaning in climate change decision-making. *Global Environmental Politics, 11*(2), 1–25. https://doi.org/10.1162/GLEP_a_00051
2. Ager, J., Fiddian-Qasmiyeh, E., & Ager, A. (2015). Local faith communities and the promotion of resilience in contexts of humanitarian crisis. *Journal of Refugee Studies, 28*(2), 202–221. https://doi.org/10.1093/jrs/fev001
3. Agnew, R. (2012). Dire forecast: A theoretical model of the impact of climate change on crime. *Theoretical Criminology, 16*(1), 21–42. https://doi.org/10.1177/1362480611416843
4. Aiena, B. J., Baczwaski, B. J., Schulenberg, S. E., & Buchanan, E. M. (2015). Measuring resilience with the RS-14: A tale of two samples. *Journal of Personality Assessment, 97*(3), 291–300. https://doi.org/10.1080/00223891.2014.951445
5. Aiena, B. J., Buchanan, E. M., Smith, C. V., & Schulenberg, S. E. (2016). Meaning, Resilience, and Traumatic Stress After the Deepwater Horizon Oil Spill: A Study of Mississippi Coastal Residents Seeking Mental Health Services. *Journal of Clinical Psychology, 72*(12), 1264–1278. https://doi.org/10.1002/jclp.22232
6. Aitsi-Selmi, A., & Murray, V. (2015). Protecting the Health and Well-being of Populations from Disasters: Health and Health Care in the Sendai Framework for Disaster Risk Reduction 2015-2030. *Prehospital and Disaster Medicine, 31*(1), 74–78. https://doi.org/10.1017/S1049023X15005531
7. Ajibade, I., Armah, F. A., Kuuire, V. Z., Luginaah, I., McBean, G., & Tenkorang, E. Y. (2015). Assessing the bio-psychosocial correlates of flood impacts in coastal areas of Lagos, Nigeria. *Journal of Environmental Planning and Management, 58*(3), 445–463. https://doi.org/10.1080/09640568.2013.861811
8. Ajibade, I., McBean, G., & Bezner-Kerr, R. (2013). Urban flooding in Lagos, Nigeria: Patterns of vulnerability and resilience among women. *Global Environmental Change, 23*(6), 1714–1725. https://doi.org/10.1016/j.gloenvcha.2013.08.009
9. Alberini, A., Chiabai, A., & Muehlenbachs, L. (2006). Using expert judgment to assess adaptive capacity to climate change: evidence from a conjoint choice survey. *Global Environmental Change, 16*(2), 123–144.https://doi.org/10.1016/j.gloenvcha.2006.02.001
10. Albrecht, G., G.M. Sartore, L. Connor, N. Higginbotham, S. Freeman, B. Kelly, H. Stain, A. Tonna, and G. Pollard, 2007: Solastalgia: The distress caused by environmental change. *Australasian Psychiatry, 15*, S95-S98. http://dx.doi.org/10.1080/10398560701701288
11. Albrecht, G. (2011). Chronic environmental change: Emerging "psychoterratic"syndromes. In Climate Change and Human Well-Being. New York: Springer. ISBN-13: 978-1441997418
12. Alderman, K., Turner, L. R., & Tong, S. (2012). Floods and human health: A systematic review. *Environment International, 47*, 37–47. https://doi.org/10.1016/j.envint.2012.06.003

13. Alderman, K., Turner, L. R., & Tong, S. (2013). Assessment of the health impacts of the 2011 summer floods in Brisbane. In Disaster medicine and public health preparedness (Vol. 7, pp. 380–386). ISBN: 1938-744X
14. Allen, J., Balfour, R., Bell, R., & Marmot, M. (2014). Social determinants of mental health. International Review of *Psychiatry, 26*(4), 392–407. DOI: 10.3109/09540261.2014.928270
15. Almedom, A. M. (2008). Resilience research and policy/practice discourse in health, social, behavioral, and environmental sciences over the last ten years. *African Health Sciences, 8*(3).
16. Almedom, A. M., Tesfamichael, B., Mohammed, Z. S., Mascie-Taylor, C. G. N., & Alemu, Z. (2007). Use of 'Sense of Coherence (SOC)'scale to measure resilience in Eritrea: Interrogating both the data and the scale. *Journal of Biosocial Science, 39*(01), 91-107. DOI:10.1017/S0021932005001112
17. Almedom, A. M., O'Byrne, D., & Jerneck, A. (2015). Principles of epistemological accountability with methodological implications for measuring, assessing, and profiling human resilience. *Ecology and Society, 20*(3). https://doi.org/10.5751/ES-07313-200309
18. Ampuero, D., Goldswosthy, S., Delgado, L. E., & Miranda J, C. (2015). Using mental well-being impact assessment to understand factors influencing well-being after a disaster. *Impact Assessment and Project Appraisal, 33*(3), 184–194. https://doi.org/10.1080/14615517.2015.1023564
19. Anderson, D. (2009). Enduring drought then coping with climate change: Lived experience and local resolve in rural mental health. *Rural Society, 19*(4), 340–352. https://doi.org/10.5172/rsj.351.19.4.340
20. Anderson, P., & Jané-Llopis, E. (2011). Mental health and global well-being. *Health Promotion International, 26*(SUPPL. 1). https://doi.org/10.1093/heapro/dar060
21. Anderson, H., Brown, C., Cameron, L. L., Christenson, M., Conlon, K. C., Dorevitch, S., ... & Hanson, A. (2017). Climate and health intervention assessment: evidence on public health interventions to prevent the negative health effects of climate change. *Climate and health technical report series. BRACE Midwest and Southeast Community of Practice. Climate and Health Program, Centers for Disease Control and Prevention*.
22. Asugeni, J., MacLaren, D., Massey, P. D., & Speare, R. (2015). Mental health issues from rising sea level in a remote coastal region of the Solomon Islands: current and future. Australasian Psychiatry: *Bulletin of Royal Australian and New Zealand College of Psychiatrists, 23*(6), 22–25. https://doi.org/10.1177/1039856215609767
23. Austin, S. E., Ford, J. D., Berrang-Ford, L., Araos, M., Parker, S., & Fleury, M. D. (2015). Public health adaptation to climate change in Canadian jurisdictions. *International Journal of Environmental Research and Public Health, 12*(1), 623–651. https://doi.org/10.3390/ijerph120100623
24. Azuma, K., Ikeda, K., Kagi, N., Yanagi, U., Hasegawa, K., & Osawa, H. (2014). Effects of water-damaged homes after flooding: health status of the residents and the environmental risk factors. *International journal of environmental health research, 24*(2), 158-175. DOI:10.1080/09603123.2013.800964

25. Bajayo, R. (2012). Building community resilience to climate change through public health planning. *Health Promotion Journal of Australia, 23*(1), 30–36.
26. Bardsley, D. K., & Wiseman, N. D. (2012). Climate change vulnerability and social development for remote indigenous communities of South Australia. *Global Environmental Change, 22*(3), 713–723. https://doi.org/10.1016/j.gloenvcha.2012.04.003
27. Barnett, J., Evans, L. S., Gross, C., Kiem, A. S., Kingsford, R. T., Palutikof, J. P., ... & Smithers, S. G. (2015). From barriers to limits to climate change adaptation: path dependency and the speed of change. *Ecology and Society*, *20*(3). http://dx.doi.org/10.5751/ES-07698-200305
28. Bei, B., C. Bryant, K.M. Gilson, J. Koh, P. Gibson, A. Komiti, H. Jackson, and F. Judd, 2013: A prospective study of the impact of floods on the mental and physical health of older adults. *Aging & Mental Health, 17*, 992-1002. http://dx.doi.org/10.1080/13607863.2013.799119
29. Bélanger, D., Gosselin, P., Valois, P., & Abdous, B. (2014). Perceived adverse health effects of heat and their determinants in deprived neighbourhoods: A cross-sectional survey of nine cities in Canada. *International Journal of Environmental Research and Public Health, 11*(11), 11028–11053. https://doi.org/10.3390/ijerph111111028
30. Bell, E. (2012). Readying health services for climate change: A policy framework for regional development. *American Journal of Public Health, 101*(5), 804–813. DOI: 10.2105/AJPH.2010.202820
31. Bergström, J., & Dekker, S. W. A. (2014). Bridging the macro and the micro by considering the meso: Reflections on the fractal nature of resilience. *Ecology and Society, 19(*4). https://doi.org/10.5751/ES-06956-190422
32. Berkes, F., & Ross, H. (2013). Community Resilience: Toward an Integrated Approach. *Society and Natural Resources, 26*(1), 5–20. https://doi.org/10.1080/08941920.2012.736605
33. Berry, H. L., Kelly, B. J., Hanigan, I. C., Coates, J. H., McMichael, A. J., Welsh, J. A., & Kjellstrom, T. (2008a). Rural mental health impacts of climate change. Commissioned report for the Garnaut Climate Change Review. Canberra: The Australian National University.
34. Berry, H. L., Kelly, B. J., Hanigan, I. C., Coates, J. H., McMichael, A. J., Welsh, J. A., & Kjellstrom, T. (2008b). Rural mental health impacts of climate change. *Commissioned report for the Garnaut Climate Change Review. Canberra: The Australian National University*.
35. Berry, H. L., & Shipley, M. (2009a). Longing to belong: personal social capital and psychological distress in an Australian coastal region. Department of Families, Housing, Community Services and Indigenous Affairs.
36. Berry, H. (2009b). Pearl in the oyster: Climate change as a mental health opportunity. *Australasian Psychiatry, 17*(6), 453–456. DOI:10.1080/10398560903045328
37. Berry, H. L., Bowen, K., & Kjellstrom, T. (2010a). Climate change and mental health: A causal pathways framework. *International Journal of Public Health, 55*, 123–132. DOI:10.1007/s00038-009-0112-0

38. Berry, H. L., Butler, J. R. A., Burgess, C. P., King, U. G., Tsey, K., Cadet-James, Y. L., … Raphael, B. (2010b). Mind, body, spirit: Co-benefits for mental health from climate change adaptation and caring for country in remote Aboriginal Australian communities. *New South Wales Public Health Bulletin, 21*(5–6), 139–145. https://doi.org/10.1071/NB10030
39. Berry, H. L., Hogan, A., Owen, J., Rickwood, D., & Fragar, L. (2011). Climate change and farmers' mental health: risks and responses. *Asia-Pacific Journal of Public Health, 23*(2), 119–132. DOI: 10.1177/1010539510392556
40. Birkmann, J., Buckle, P., Jaeger, J., Pelling, M., Setiadi, N., Garschagen, M., … Kropp, J. (2010). Extreme events and disasters: A window of opportunity for change? Analysis of organizational, institutional and political changes, formal and informal responses after mega-disasters. *Natural Hazards, 55*(3), 637–655. https://doi.org/10.1007/s11069-008-9319-2
41. Bonanno, G. A. (2012). Uses and abuses of the resilience construct: Loss, trauma, and health-related adversities. *Social Science & Medicine, 74*(5), 753-756. DOI:10.1016/j.socscimed.2011.11.022
42. Bourque, F., & Cunsolo Willox, A. (2014). Climate change: The next challenge for public mental health? *International Review of Psychiatry, 26*(4), 415–422.
43. Bowles, D. C. (2015). Climate change and health adaptation: Consequences for indigenous physical and mental health. *Annals of Global Health, 81*(3), 427–431. https://doi.org/10.1016/j.aogh.2015.06.004
44. Bragg, E., & Reser, J. (2012). Ecopsychology in the antipodes: Perspectives from Australia and New Zealand. *Ecopsychology, 4*(4), 253–265. https://doi.org/10.1089/eco.2012.0085
45. Brown, K. (2015). *Resilience, development and global change*. Routledge.
46. Brown, K., & Westaway, E. (2011). Agency, capacity, and resilience to environmental change: lessons from human development, well-being, and disasters. *Annual Review of Environment and Resources, 36*(1), 321. https://doi.org/10.1146/annurev-environ-052610-092905
47. Bunch, M. J., Morrison, K. E., Parkes, M. W., & Venema, H. D. (2011). Promoting health and well-being by managing for social–ecological resilience: the potential of integrating ecohealth and water resources management approaches. *Ecology and Society*, *16*(1).
48. Bunyavanich, S., Landrigan, C. P., McMichael, A. J., & Epstein, and P. R. (2003). The impact of climate change on child health. *Ambulatory, 3*(1), 44–52. https://doi.org/10.1367/1539-4409(2003)003<0044:TIOCCO>2.0.CO;2
49. Burch, S., Shaw, A., Dale, A., & Robinson, J. (2014). Triggering transformative change: A development path approach to climate change response in communities. *Climate Policy, 14*(4), 467-487. doi:10.1080/14693062.2014.876342
50. Burch, S. (2010). Transforming barriers into enablers of action on climate change: Insights from three municipal case studies in British Columbia, Canada. *Global Environmental Change, 20*(2), 287-297. doi:10.1016/j.gloenvcha.2009.11.009
51. Burton, H., Rabito, F., Danielson, L., & Takaro, T. K. (2016). Health effects of flooding in Canada: A 2015 review and description of gaps in research. Canadian *Water Resources Journal, 41*(1–2), 238–249. https://doi.org/10.1080/07011784.2015.1128854

52. Butler, C. D., Bowles, D. C., McIver, L., & Page, L. (2014). 26 Mental Health, Cognition and the Challenge of Climate Change. *Climate change and global health*, 251.
53. Berkes, F. and D. Jolly. 2001. Adapting to climate change: Social-ecological resilience in a Canadian western arctic community.*Conservation Ecology 5* (2): 18–39.
54. Cameron, E. S. (2012). Securing indigenous politics: A critique of the vulnerability and adaptation approach to the human dimensions of climate change in the Canadian arctic. *Global Environmental Change, 22*(1), 103–114. https://doi.org/10.1016/j.gloenvcha.2011.11.004
55. Cardwell, F. S., & Elliott, S. J. (2013). Making the links: do we connect climate change with health? A qualitative case study from Canada. *BMC Public Health, 13*(1), 1. DOI: 10.1186/1471-2458-13-208
56. Carnie, T.-L., Berry, H. L., Blinkhorn, S. A., & Hart, C. R. (2011). In their own words: Young people's mental health in drought-affected rural and remote NSW. *Australian Journal of Rural Health, 19*(5), 244–248. https://doi.org/10.1111/j.1440-1584.2011.01224.x
57. Carroll, B., Balogh, R., Morbey, H., & Araoz, G. (2010). Health and social impacts of a flood disaster: responding to needs and implications for practice. *Disasters, 34*(4), 1045–1063. DOI:10.1111/j.1467-7717.2010.01182.x
58. Carroll, B., Morbey, H., Balogh, R., & Araoz, G. (2009). Flooded homes, broken bonds, the meaning of home, psychological processes and their impact on psychological health in a disaster. *Health & Place, 15(*2), 540–547. https://doi.org/10.1016/j.healthplace.2008.08.009
59. Castleden, M., McKee, M., Murray, V., & Leonardi, G. (2011). Resilience thinking in health protection. *Journal of Public Health, 33*(3), 369–377. DOI:10.1093/pubmed/fdr027
60. Chand, P. K., & Murthy, P. (2008). Climate change and mental health. In Regional Health Forum (Vol. 12, pp. 43–48).
61. Cheng, J. J., & Berry, P. (2013). Health co-benefits and risks of public health adaptation strategies to climate change: A review of current literature. *International Journal of Public Health, 58*(2), 305–311. https://doi.org/10.1007/s00038-012-0422-5
62. Cheshire, L., Esparcia, J., & Shucksmith, M. (2015). Community resilience, social capital and territorial governance [Resiliencia comunitaria, capital social y gobernanza territorial]. *Ager*, (18), 7–38. https://doi.org/10.4422/ager.2015.08
63. Chowdhury, P. D., Haque, C. E., & Smith, G. (2011). A critical review of climate change induced psychosocial impacts and options for strengthening social support systems. *American Journal of Environmental Sciences*, *7*(4), 316. DOI: 10.3844/ajessp.2011.316.330
64. Cameron, E., Mearns, R., & McGrath, J. T. (2015). Translating climate change: Adaptation, resilience, and climate politics in Nunavut, Canada. *Annals of the Association of American Geographers, 105*(2), 274-283. https://doi.org/10.1080/00045608.2014.973006
65. Clarke, K. L., & Berry, P. (2012). From theory to practice: a Canadian case study of the utility of climate change adaptation frameworks to address health impacts. *International Journal of Public Health, 57*(1), 167–174. DOI: 10.1007/s00038-011-0292-2

66. Clayton, S., Manning, C. M., & Hodge C. (2014). Beyond storms & droughts: The psychological impacts of climate change. Washington, DC: American Psychological Association and ecoAmerica.
67. Clayton, S., Manning, C. M., Krygsman, K., & Speiser, M. (2017). Mental Health and Our Changing Climate: Impacts, Implications, and Guidance. Washington, D.C.: American Psychological Association, and ecoAmerica.
68. Congues, J. M. (2014). Promoting collective well-being as a means of defying the odds: Drought in the Goulburn Valley, Australia. *Rural Society, 20*(3), 229–242. https://doi.org/10.1080/10371656.2014.11082067
69. Coombe, J., Mackenzie, L., Munro, R., Hazell, T., Perkins, D., & Reddy, P. (2015). Teacher-mediated interventions to support child mental health following a disaster: A systematic review. *PLoS Currents, 7*(DISASTERS). https://doi.org/10.1371/currents.dis.466c8c96d879e2663a1e5e274978965d
70. Costello, A., Abbas, M., Allen, A., Ball, S., Bell, S., Bellamy, R., … Patterson, C. (2009). Managing the health effects of climate change. Lancet and University College London Institute for Global Health Commission. *The Lancet, 373*(9676), 1693–1733. https://doi.org/10.1016/S0140-6736(09)60935-1
71. Cox, R. S., & Hamlen, M. (2015). Community disaster resilience and the rural resilience index. *American Behavioral Scientist*, *59*(2), 220-237. https://doi.org/10.1177/0002764214550297
72. Crabtree, A. (2012). Climate change and mental health following flood disasters in developing countries, A review of the epidemiological literature: What do we know, what is being recommended? *Australasian Journal of Disaster and Trauma Studies, 12*(1), 21–30.
73. Crabtree, A. (2013). Questioning Psychosocial Resilience After Flooding and the Consequences for Disaster Risk Reduction. *Social Indicators Research, 113(*2), 711–728. https://doi.org/10.1007/s11205-013-0297-8
74. Cunsolo Willox, A., Harper, S. L., Ford, J. D., Landman, K., Houle, K., & Edge, V. L. (2012). "From this place and of this place:" Climate change, sense of place, and health in Nunatsiavut, Canada. *Social Science & Medicine, 75*(3), 538–547. DOI:10.1016/j.socscimed.2012.03.043
75. Cunsolo Willox, A. C., Harper, S. L., Edge, V. L., Landman, K., Houle, K., & Ford, J. D. (2013a). The land enriches the soul: On climatic and environmental change, affect, and emotional health and well-being in Rigolet, Nunatsiavut, Canada. *Emotion Space and Society*, *6*, 14-24. https://doi.org/10.1016/j.emospa.2011.08.005
76. Cunsolo Willox, A., Harper, S. L., & Ford, J. D. (2013b). Climate change and mental health: An exploratory case study from Rigolet. Nunatsiavut: Canada. *Climatic Change*, *121*(2), 255-270.
77. Cunsolo Willox, A., Stephenson, E., Allen, J., Bourque, F., Drossos, A., Elgarøy, S., … Wexler, L. (2014). Examining relationships between climate change and mental health in the Circumpolar North. *Regional Environmental Change, 15*(1), 169–182. https://doi.org/10.1007/s10113-014-0630-z
78. Cusack, L., de Crespigny, C., & Athanasos, P. (2011). Heatwaves and their impact on people with alcohol, drug and mental health conditions: a discussion paper on clinical practice considerations. *Journal of Advanced Nursing, 67*(4), 915–922. DOI:10.1111/j.1365-2648.2010.05551.x

79. Cutter, S.L., Barnes, L., Berry, M., Burton, C.G., Evans, E., Tate, E.C., Webb, J., 2008.Community and regional resilience: perspectives from hazards, disasters, and emergency management. In: CARRI Research Report 1 Community and Regional Resilience Institute, Oak Ridge, TN.
80. Davidson, J. R. T., & McFarlane, A. C. (2006). The extent and impact of mental health problems after disaster. *Journal of Clinical Psychiatry, 67*, 9–14.
81. Dean, J. G., & Stain, H. J. (2010). Mental health impact for adolescents living with prolonged drought. *Australian Journal of Rural Health, 18*(1), 32–37. DOI: 10.1111/j.1440-1584.2009.01107.x
82. Dean, J., van Dooren, K., & Weinstein, P. (2011). Does biodiversity improve mental health in urban settings? *Medical Hypotheses, 76*(6), 877–880. https://doi.org/10.1016/j.mehy.2011.02.040
83. Deeming, H., Fordham, M., & Swartling, Å. G. (2014). Resilience and adaptation to hydrometeorological hazards. *Hydrometeorological Hazards*, 291-316. https://doi.org/10.1002/9781118629567.ch4b
84. Deppisch, S., & Hasibovic, S. (2013). Social-ecological resilience thinking as a bridging concept in transdisciplinary research on climate-change adaptation. *Natural Hazards, 67*(1), 117–127. https://doi.org/10.1007/s11069-011-9821-9
85. Dodgen, D., D. Donato, N. Kelly, A. La Greca, J. Morganstein, J. Reser, J. Ruzek, S. Schweitzer, M.M. Shimamoto, K. Thigpen Tart, and R. Ursano, 2016: Ch. 8: Mental Health and Well-Being. The Impacts of Climate Change on Human Health in the United States: A Scientific Assessment. U.S. Global Change Research Program, Washington, DC, 217–246. http://dx.doi.org/10.7930/J0TX3C9H
86. Doherty, T. J., & Clayton, S. (2011). The psychological impacts of global climate change. *American Psychologist, 66*(265–276), 10–1037. http://dx.doi.org/10.1037/a0023141
87. Dorji, C. (2006). Mental health and psychosocial aspects of disaster preparedness in Bhutan. *International Review of Psychiatry, 18*(6), 537–546. DOI:10.1080/09540260601037979
88. Douglas, E. M., Kirshen, P. H., Paolisso, M., Watson, C., Wiggin, J., Enrici, A., & Ruth, M. (2012). Coastal flooding, climate change and environmental justice: identifying obstacles and incentives for adaptation in two metropolitan Boston Massachusetts communities. Mitigation and Adaptation Strategies for Global. *Change, 17*(5), 537–562. https://doi.org/10.1007/s11027-011-9340-8
89. Durkalec, A., Furgal, C., Skinner, M. W., & Sheldon, T. (2015). Climate change influences on environment as a determinant of Indigenous health: Relationships to place, sea ice, and health in an Inuit community. *Social Science and Medicine,* 136–137, 17–26. https://doi.org/10.1016/j.socscimed.2015.04.026
90. Ebi, K. L., & Bowen, K. (2016). Extreme events as sources of health vulnerability: Drought as an example. *Weather and Climate Extremes, 11,* 95–102. https://doi.org/10.1016/j.wace.2015.10.001
91. Edwards, B., Gray, M., & Hunter, B. (2015). The Impact of Drought on Mental Health in Rural and Regional Australia. *Social Indicators Research, 121*(1), 177–194. https://doi.org/10.1007/s11205-014-0638-2

92. Edwards, T., & Wiseman, J. (2011). Climate change, resilience and transformation: challenges and opportunities for local communities. In Climate Change and Human Well-Being. New York: Springer.
93. Eichler, M. (2015). Raising children to cope with climate change?. Ca*nadian Review of Sociology/Revue canadienne. De, 52*(2), 232–240. https://doi.org/10.1111/cars.12075
94. Eisenman, D., McCaffrey, S., Donatello, I., & Marshal, G. (2015). An Ecosystems and Vulnerable Populations Perspective on Solastalgia and Psychological Distress After a Wildfire. *EcoHealth, 12*(4), 602–610. https://doi.org/10.1007/s10393-015-1052-1
95. Evans, L. S., Hicks, C. C., Adger, W. N., Barnett, J., Perry, A. L., Fidelman, P., & Tobin, R. (2016). Structural and psycho-social limits to climate change adaptation in the great barrier reef region. *PloS, 11*(3), 150575. https://doi.org/10.1371/journal.pone.0150575
96. Every-Palmer, S., Mcbride, S., Berry, H., & Menkes, D. B. (2016). Climate change and psychiatry. *Australian and New Zealand Journal of Psychiatry, 50*(1), 16–18. https://doi.org/10.1177/0004867415615946
97. Fernandez, A., Black, J., Jones, M., Wilson, L., Salvador-Carulla, L., Astell-Burt, T., & Black, D. (2015). Flooding and mental health: A systematic mapping review. *PLoS ONE, 10*(4). https://doi.org/10.1371/journal.pone.0119929
98. Few, R., Ahern, M., Matthies, F., & Kovats, S. (2004). Floods, health and climate change: a strategic review. Norwich: Tyndall Centre for Climate Change Research.
99. Fleming, A., Dowd, A.-M., Gaillard, E., Park, S., & Howden, M. (2015). “Climate change is the least of my worries”: Stress limitations on adaptive capacity. *Rural Society, 24*(1), 24–41. https://doi.org/10.1080/10371656.2014.1001481
100. Ford, J. D. (2012). Indigenous health and climate change. *American Journal of Public Health, 102*(7), 1260–1266. https://doi.org/10.2105/AJPH.2012.300752
101. Ford, J. D., Stephenson, E., Cunsolo Willox, A., Edge, V., Farahbakhsh, K., Furgal, C., … Sherman, M. (2016). Community-based adaptation research in the Canadian Arctic. Wiley Interdisciplinary Reviews: *Climate Change, 7*(2), 175–191. https://doi.org/10.1002/wcc.376
102. Fresque-Baxter, J. A., & Armitage, D. (2012). Place identity and climate change adaptation: A synthesis and framework for understanding. Wiley Interdisciplinary Reviews: *Climate Change, 3(*3), 251–266. https://doi.org/10.1002/wcc.164
103. Friel, S., Berry, H., Dinh, H., O’Brien, L., & Walls, H. L. (2014). The impact of drought on the association between food security and mental health in a nationally representative Australian sample. *BMC Public Health, 14*, 1102. https://doi.org/10.1186/1471-2458-14-1102
104. Fritze, J. G., Blashki, G. A., Burke, S., & Wiseman, J. (2008). Hope, despair and transformation: Climate change and the promotion of mental health and wellbeing. *International Journal of Mental Health Systems, 2*, 1–13. https://doi.org/10.1186/1752-4458-2-13

105. Fundter, D. Q., Jonkman, B., Beerman, S., Goemans, C. L., Briggs, R., Coumans, F., & Bierens, J. (2008). Health impacts of large-scale floods: governmental decision-making and resilience of the citizens. *Prehospital and Disaster Medicine, 23*(2), 70–73.https://doi.org/10.1017/S1049023X00021282
106. Furgal, C., Buell, M., Chan, L., Edge, V., Martin, D., & Ogden, N. (2008). Health impacts of climate change in Canada's North. Human health in a changing climate: a Canadian assessment of vulnerabilities and adaptive capacity. Health Canada, Ottawa.
107. Füssel, H. M. (2007). Vulnerability: a generally applicable conceptual framework for climate change research. *Global environmental change, 17*(2), 155-167. https://doi.org/10.1016/j.gloenvcha.2006.05.002
108. Galea, S., Brewin, C. R., Gruber, M., Jones, R. T., King, D. W., King, L. A., ... & Kessler, R. C. (2007). Exposure to hurricane-related stressors and mental illness after Hurricane Katrina. *Archives of general psychiatry*, *64*(12), 1427-1434. DOI:10.1001/archpsyc.64.12.1427
109. Gibbs, L., Waters, E., Bryant, R. A., Pattison, P., Lusher, D., Harms, L., … Forbes, D. (2013). Beyond Bushfires: Community, Resilience and Recovery - A longitudinal mixed method study of the medium to long term impacts of bushfires on mental health and social connectedness. *BMC Public Health, 13*(1). https://doi.org/10.1186/1471-2458-13-1036
110. Gifford, R. (2011). The dragons of inaction: Psychological barriers that limit climate change mitigation and adaptation. *American Psychologist, 66*, 290-302. DOI:10.1037/a0023566
111. Goh, A. H. (2012). A literature review of the gender-differentiated impacts of climate change on women's and men's assets and well-being in developing countries. International Food Policy Research Institute, CAPRi Work.
112. Gray, S. (2008). Long-term health effects of flooding. *Journal of Public Health, 30*(4), 353–354. https://doi.org/10.1093/pubmed/fdn092
113. Green, D., & Minchin, L. (2014). Living on climate-changed country: Indigenous health, well-being and climate change in remote Australian communities. *EcoHealth, 11*(2), 263–272. https://doi.org/10.1007/s10393-013-0892-9
114. Green, D., Niall, S., & Morrison, J. (2012). Bridging the gap between theory and practice in climate change vulnerability assessments for remote Indigenous communities in northern Australia. *Local Environment, 17*(3), 295–315. https://doi.org/10.1080/13549839.2012.665857
115. Greene, G., Paranjothy, S., & Palmer, S. R. (2015). Resilience and vulnerability to the psychological harm from flooding: The role of social cohesion. *American Journal of Public Health, 105*(9), 1792–1795. https://doi.org/10.2105/AJPH.2015.302709
116. Greene, R. R. (2014). Resilience as Effective Functional Capacity: An Ecological-Stress Model. *Journal of Human Behavior in the Social Environment, 24*(8), 937–950. https://doi.org/10.1080/10911359.2014.921589
117. Gruebner, O., Lowe, S. R., Sampson, L., & Galea, S. (2015). The geography of post-disaster mental health: Spatial patterning of psychological vulnerability and resilience factors in New York City after Hurricane Sandy. *International Journal of Health Geographics, 14*(1). https://doi.org/10.1186/s12942-015-0008-6

118. Gunn, K. M., Kettler, L. J., Skaczkowski, G. L. A., & Turnbull, D. A. (2012). Farmers' stress and coping in a time of drought. *Rural and Remote Health*, 12(4).
119. Gutierrez, K. S., & LePrevost, C. E. (2016). Climate justice in rural southeastern united states: A review of climate change impacts and effects on human health. *International Journal of Environmental Research and Public Health, 13*(2). https://doi.org/10.3390/ijerph13020189
120. Hanigan, I. C., Butler, C. D., Kokic, P. N., & Hutchinson, M. F. (2012). Suicide and drought in New South Wales, Australia, 1970–2007. In Proceedings of the National Academy of Sciences (Vol. 109, pp. 13950–13955).
121. Hannigan, B., & Coffey, M. (2011). Where the wicked problems are: The case of mental health. *Health Policy, 101*(3), 220–227. https://doi.org/10.1016/j.healthpol.2010.11.002
122. Harper, S. L., Edge, V. L., Ford, J., Willox, A. C., Wood, M., McEwen, S. A., … Namanya, D. B. (2015). Climate-sensitive health priorities in Nunatsiavut, Canada. *BMC Public Health, 15*(1). https://doi.org/10.1186/s12889-015-1874-3
123. Hart, C. R., Berry, H. L., & Tonna, A. M. (2011). Improving the mental health of rural New South Wales communities facing drought and other adversities. *Australian Journal of Rural Health, 19*(5), 231–238. https://doi.org/10.1111/j.1440-1584.2011.01225.x
124. Henderson, S., & Mulder, R. (2015). Climate change and mental disorders. *Australian and New Zealand Journal of Psychiatry, 49*(11), 1061–1062. https://doi.org/10.1177/0004867415610639
125. Höfler, M. (2014). Psychological Resilience Building in Disaster Risk Reduction: Contributions from Adult Education. *International Journal of Disaster Risk Science, 5*(1), 33–40. https://doi.org/10.1007/s13753-014-0009-2
126. Holmes, T. (2015). Community Risk and Resilience to Climate Hazards and Extreme. In Events in the Turtle Region of Trinidad.
127. Holmgren, D. (2012). Future scenarios: how communities can adapt to peak oil and climate change. Chelsea: Green Publishing.
128. Hunter, E. (2009). "Radical hope" and rain: Climate change and the mental health of Indigenous residents of northern Australia. *Australasian Psychiatry, 17*(6), 445–452. https://doi.org/10.1080/10398560903062927
129. Hutton, D. (2005). Psychosocial Aspects of Climate Change in Canada:A Review of Current Literature and Research Recommendations. Health Canada.
130. Imperiale, A. J., & Vanclay, F. (2016). Experiencing local community resilience in action: Learning from post-disaster communities. *Journal of Rural Studies, 47*, 204–219. https://doi.org/10.1016/j.jrurstud.2016.08.002
131. Inder, K. J., Berry, H., & Kelly, B. J. (2011). Using cohort studies to investigate rural and remote mental health. *Australian Journal of Rural Health, 19*(4), 171–178. https://doi.org/10.1111/j.1440-1584.2011.01208.x
132. Jain, V. (2015). The Post-2015 Sustainable Development Agenda—Are We Doing Mental Health Justice? *Annals of Global Health, 81*(3), 461–462. https://doi.org/10.1016/j.aogh.2015.06.009

133. Jane-Llopis, E., Anderson, P., Stewart-Brown, S., Weare, K., Wahlbeck, K., McDaid, D., … Litchfield, P. (2011). Reducing the silent burden of impaired mental health. *Journal of Health Communication, 16*(SUPPL. 2), 59–74. https://doi.org/10.1080/10810730.2011.601153
134. Jaspal, R., Nerlich, B., & Cinnirella, M. (2014). Human responses to climate change: Social representation, identity and socio-psychological action. *Environmental Communication, 8*(1), 110–130. https://doi.org/10.1080/17524032.2013.846270
135. Jenkins, R., Baingana, F., Ahmad, R., McDaid, D., & Atun, R. (2011). International and national policy challenges in mental health. *Mental Health in Family Medicine, 8*(2), 101.
136. Joerin, J., Ramasamy, R., Krishnamurthy, R. (2014).The concept of resilience to disasters. Community, Environment and *Disaster Risk Management, 15*, 35–48. https://doi.org/10.1108/S2040-726220140000015002
137. Jones, M. K., Wootton, B. M., Vaccaro, L. D., & Menzies, R. G. (2012). The impact of climate change on obsessive compulsive checking concerns. *Australian and New Zealand Journal of Psychiatry, 46*(3), 265–270. DOI:10.1177/0004867411433951
138. Keim, M. E. (2008). Building Human Resilience. The Role of Public Health Preparedness and Response as an Adaptation to *Climate Change. American Journal of Preventive Medicine, 35*(5), 508–516. https://doi.org/10.1016/j.amepre.2008.08.022
139. Keim, M. E. (2011). Preventing disasters: Public health vulnerability reduction as a sustainable adaptation to climate change. *Disaster Medicine and Public Health Preparedness, 5*(2), 140–148. https://doi.org/10.1001/dmp.2011.30
140. Keller, R. C. (2013). Place matters: Mortality, space, and urban form in the 2003 Paris heat wave disaster. *French Historical Studies, 36*(2), 299-330. https://doi.org/10.1215/00161071-1960682
141. Kessler, R. C., Galea, S., Gruber, M. J., Sampson, N. A., Ursano, R. J., & Wessely, and S. (2008). Trends in mental illness and suicidality after Hurricane Katrina. *Molecular Psychiatry, 13*, 374–384. https://doi.org/doi:10.1038
142. Kirmayer, L. J., Dandeneau, S., Marshall, E., Phillips, M. K., & Williamson, K. J. (2011). Rethinking resilience from indigenous perspectives. *Canadian Journal of Psychiatry, 56*(2), 84–91. DOI:10.1177/070674371105600203
143. Kjellstrom, T., & McMichael, A. J. (2013). Climate change threats to population health and well-being: The imperative of protective solutions that will last. *Global Health Action, 6*(1). https://doi.org/10.3402/gha.v6i0.20816
144. Koger, S. M., Leslie, K. E., & Hayes, E. D. (2011). Climate change: Psychological solutions and strategies for change. *Ecopsychology, 3*(4), 227–235. https://doi.org/10.1089/eco.2011.0041
145. Kuehne, G. (2014). How do farmers' climate change beliefs affect adaptation to climate change? *Society & Natural Resources, 27*(5), 492–506. https://doi.org/10.1080/08941920.2013.861565
146. Natalia, K. (2011). Climate change effects on human health in a gender perspective: some trends in Arctic research. *Global Health Action*, *4*(1), 7913. DOI: 10.3402/gha.v4i0.7913
147. LaLone, M. B. (2012). Neighbors helping neighbors: An examination of the social capital mobilization process for community resilience to environmental disasters. *Journal of Applied Social Science, 6*(2), 209-237.

148. Lamond, J. (2014). The role of flood memory in the impact of repeat flooding on mental health. *WIT Transactions on Ecology and the Environment, 184*, 187–199. https://doi.org/10.2495/FRIAR140161
149. Lang, C. (2015). Climate change and mental health. *Psychiatric Times*, *32*(10), 1-1.
150. Leff, J. (2008). Climate change in psychiatry: periodic fluctuations or terminal trend. *Society and, psychosis, 11*. ISBN: 978-052-68959-5
151. Leslie, P., & McCabe, J. T. (2013). Response diversity and resilience in social-ecological systems. *Current Anthropology, 54(*2), 114.
152. Levine, S., Ludi, E., & Jones, L. (2011). Rethinking support for adaptive capacity to climate change. In Oxfam Policy and Practice: Climate Change and Resilience (Vol. 7, pp. 49–97).
153. Levy, B. S., & Patz, J. A. (2015). Climate change, human rights, and social justice. *Annals of Global Health, 81(*3), 310–322. https://doi.org/10.1016/j.aogh.2015.08.008
154. Lindahl, K. K., Balajee, S. S., & Wiggins, N. (2013). Complete health equity: Bringing mental health into the equity dialogue. In *Social Sustainability* (pp. 176-200). Routledge.
155. Loboda, T. V. (2014b). Adaptation strategies to climate change in the Arctic: A global patchwork of reactive community-scale initiatives. *Environmental Research Letters, 9*(11). https://doi.org/10.1088/1748-9326/9/11/111006
156. Lorenz, D. F., & Dittmer, C. (2016). Resilience in catastrophes, disasters and emergencies. In *New perspectives on resilience in socio-economic spheres* (pp. 25-59). Springer VS, Wiesbaden.
157. Lowe, D., Ebi, K.L., Forsberg, B. (2013). Factors increasing vulnerability to health effects before, during, and after floods. *Int. J. Environ. Res. Public Health 10*, 7015-7067. doi: 10.3390/ijerph10127015
158. Luber, G., & Lemery, J. (2015). Global climate change and human health: From science to practice. John Wiley & Sons. Chapter. 10 Climate change and population mental health by Abdulrahman M. El Sayed, and Sandro Galea
159. MacDonald, J. P., Willox, A. C., Ford, J. D., Shiwak, I., Wood, M., & Team, I. M. H. A. C. C. (2015). Protective factors for mental health and well-being in a changing climate: Perspectives from Inuit youth in Nunatsiavut, Labrador. *Social Science & Medicine, 141*, 133–141. https://doi.org/10.1016/j.socscimed.2015.07.017
160. Manyena, S. B. (2014). Disaster resilience: A question of "multiple faces" and "multiple spaces"? *International Journal of Disaster Risk Reduction, 8*, 1–9. https://doi.org/10.1016/j.ijdrr.2013.12.010
161. Marinucci, G. D., Luber, G., Uejio, C. K., Saha, S., & Hess, J. J. (2014). Building resilience against climate effects—A novel framework to facilitate climate readiness in public health agencies. *International journal of environmental research and public health, 11*(6), 6433-6458.
162. MacDonald, J. P., Harper, S. L., Willox, A. C., & Edge, V. L. (2013). A necessary voice: Climate change and lived experiences of youth in Rigolet, Nunatsiavut, Canada. *Global Environmental Change*, *23*(1), 360-371.https://doi.org/10.1016/j.gloenvcha.2012.07.010

163. McFarlane, A. C., & Williams, R. (2012). Mental health services required after disasters: Learning from the lasting effects of disasters. In *Depression research and treatment, 2012*, http://dx.doi.org/10.1155/2012/970194
164. Miles, S.B., Chang, S.E., 2011. ResilUS: a community based disaster resiliencemodel. Cartogr. Geogr. *Inform. Sci. 38*, 36 https://doi.org/10.1559/1523040638136
165. Molnar, J. J. (2010). Climate change and societal response: Livelihoods, communities, and the environment. *Rural Sociology, 75*(1), 1–16. https://doi.org/10.1111/j.1549-0831.2010.00011.x
166. Motesharrei, S., Rivas, J., Kalnay, E., Asrar, G. R., Busalacchi, A. J., Cahalan, R. F., ... & Hubacek, K. (2016). Modeling sustainability: population, inequality, consumption, and bidirectional coupling of the Earth and Human Systems. *National Science Review*, *3*(4), 470-494. https://doi.org/10.1093/nsr/nww081
167. Morello-Frosch, R., Brown, P., Lyson, M., Cohen, A., & Krupa, K. (2011). Community voice, vision, and resilience in post-Hurricane Katrina recovery. *Environmental Justice, 4*(1), 71-80. https://doi.org/10.1089/env.2010.0029
168. Morrissey, S. A., & Reser, J. P. (2007). Natural disasters, climate change and mental health considerations for rural Australia. *Australian Journal of Rural Health, 15*(2), 120–125. https://doi.org/10.1111/j.1440-1584.2007.00865.x
169. Morss, R. E., Wilhelmi, O. V., Meehl, G. A., & Dilling, L. (2011). Improving societal outcomes of extreme weather in a changing climate: an integrated perspective. *Annual Review of Environment and Resources, 36*, 1-25. https://doi.org/10.1146/annurev-environ-060809-100145
170. Moser, S. C. (2013). Navigating the political and emotional terrain of adaptation: Community engagement when climate change comes home. *Successful adaptation to climate change: Linking science and policy in a rapidly changing world*, 289-305.
171. Munro, A., Kovats, R. S., Rubin, G. J., Waite, T. D., Bone, A., Armstrong, B., ... & Oliver, I. (2017). Effect of evacuation and displacement on the association between flooding and mental health outcomes: a cross-sectional analysis of UK survey data. *The Lancet Planetary Health*, *1*(4), e134-e141. doi: 10.1016/S2542-5196(17)30047-5
172. Myers, S. S., & Bernstein, A. (2011). The coming health crisis: Indirect health effects of global climate change. *F1000 Biol Rep, 3*(1), 3. doi: 10.3410/B3-3
173. Myers, T.A., M.C. Nisbet, E.W. Maibach, and A.A.Leiserowitz, 2012: A public health frame arouses hopeful emotions about climate change. *Climatic Change, 113*, 1105-1112. http://dx.doi.org/10.1007/s10584-012-0513-6
174. Nahar, N., Blomstedt, Y., Wu, B., Kandarina, I., Trisnantoro, L., & Kinsman, J. (2014). Increasing the provision of mental health care for vulnerable, disaster-affected people in Bangladesh. *BMC Public Health, 14*(1). https://doi.org/10.1186/1471-2458-14-708
175. Neria, Y., Galea, S., & Norris, F. H. (Eds.). (2009). Mental health and disasters. Cambridge University Press.
176. Neria, Y., & Shultz, J. M. (2012). Mental health effects of Hurricane Sandy: characteristics, potential aftermath, and response. *Jama, 308*(24), 2571–2572. 10.1001/jama.2012.110700

177. North, C. S., & Pfefferbaum, B. (2013). Mental health response to community disasters: a systematic review. *Jama, 310*(5), 507-518. doi: 10.1001/jama.2013.107799.
178. Nurse, J., Basher, D., Bone, A., & Bird, W. (2010). An ecological approach to promoting population mental health and well-being - A response to the challenge of climate change. *Perspectives in Public Health*, 130(1), 27–33. https://doi.org/10.1177/1757913909355221
179. Oba, N., Suntayakorn, C., Sangkaewsri, R., Longchupol, C., Lohitpintu, I., & Kumsri, T. (2010). The enhancement of adaptation and psychological well-being among victims of flooding and landslide in Thailand. *Journal of the Medical Association of Thailand, 93*(3), 351–357.
180. O'Brien, K., Clair, A. L. S., & Kristoffersen, B. (Eds.). (2010). Climate change, ethics and human security. Cambridge University Press.
181. OBrien, L. V., Berry, H. L., Coleman, C., & Hanigan, I. C. (2014). Drought as a mental health exposure. *Environmental Research, 131*, 181–187. https://doi.org/10.1016/j.envres.2014.03.014
182. O'Donnell, M. L., & Forbes, D. (2016). Natural disaster, older adults, and mental health-a dangerous combination. *International Psychogeriatrics, 28*(1), 9–10. https://doi.org/10.1017/S1041610215001891
183. Ojala, M. (2012). How do children cope with global climate change? Coping strategies, engagement, and well-being. *Journal of Environmental Psychology, 32*(3), 225–233. https://doi.org/10.1016/j.jenvp.2012.02.004
184. Ojala, M. (2013). Coping with climate change among adolescents: Implications for subjective well-being and environmental engagement. *Sustainability, 5(*5), 2191–2209. https://doi.org/10.3390/su5052191
185. Ojala, M. (2016). Preparing children for the emotional challenges of climate change. *Education in Times of Environmental Crises: Teaching Children to Be Agents of Change*, 210.
186. Opara, C. C. (2014). Climate change phenomenon: Causes, consequences, mitigation and some adaptation strategies in Nigeria. In The Impact of Climate Change on Sub-Saharan Africa: Case Studies in Cameroon, Nigeria and UgandaVolume 6, Pages 13-36. ISBN: 978-365304584-0;978-363165347-0
187. Ortega-Egea, J. M., García-de-Frutos, N., & Antolín-López, R. (2014). Why do some people do "more" to mitigate climate change than others? Exploring heterogeneity in psycho-social associations. *PLoS One, 9*(9), e106645. https://doi.org/10.1371/journal.pone.0106645
188. Ostry, A., Ogborn, M., Bassil, K. L., Takaro, T. K., & Allen, D. M. (2010). Climate change and health in British Columbia: Projected impacts and a proposed agenda for adaptation research and policy. *International Journal of Environmental Research and Public Health, 7*(3), 1018–1035. https://doi.org/10.3390/ijerph7031018
189. Padhy, S. K., Sarkar, S., Panigrahi, M., & Paul, S. (2015). Mental health effects of climate change. *Indian Journal of Occupational and Environmental Medicine, 19*(1), 3–7. https://doi.org/10.4103/0019-5278.156997
190. Page, L. A., & Howard, L. M. (2010). The impact of climate change on mental health (but will mental health be discussed at Copenhagen?). *Psychological Medicine, 40*(2), 177–180.

191. Parlee, B., & Furgal, C. (2012). Well-being and environmental change in the arctic: A synthesis of selected research from Canada's International Polar Year program. *Climatic Change, 115*(1), 13–34. https://doi.org/10.1007/s10584-012-0588-0
192. Parsons, M., Glavac, S., Hastings, P., Marshall, G., McGregor, J., McNeill, J., … Stayner, R. (2016). Top-down assessment of disaster resilience: A conceptual framework using coping and adaptive capacities. *International Journal of Disaster Risk Reduction, 19*, 1–11. https://doi.org/10.1016/j.ijdrr.2016.07.005
193. Phua, K.-L. (2015). Redesigning healthcare systems to meet the health challenges associated with climate change in the twenty-first century. *Journal of Emergency Management, 13*(3), 255–263. https://doi.org/10.5055/jem.2015.0239
194. Polain, J. D., Berry, H. L., & Hoskin, J. O. (2011). Rapid change, climate adversity and the next "big dry": Older farmers' mental health. *Australian Journal of Rural Health, 19*(5), 239–243. https://doi.org/10.1111/j.1440-1584.2011.01219.x
195. Popovski, V., & Mundy, K. G. (2012). Defining climate-change victims. *Sustainability Science, 7*(1), 5–16. https://doi.org/10.1007/s11625-011-0138-0
196. Powers, J. R., Dobson, A. J., Berry, H. L., Graves, A. M., Hanigan, I. C., & Loxton, D. (2015). Lack of association between drought and mental health in a cohort of 45-61 year old rural Australian women. *Australian and New Zealand Journal of Public Health, 39*(6), 518–523. https://doi.org/10.1111/1753-6405.12369
197. Rahman, M. S., Mohamad, O. B., & Zarim, Z. (2014). Climate change: a review of its health impact and perceived awareness by the young citizens. *Global Journal of Health Science, 6*(4), 196–204. https://doi.org/10.5539/gjhs.v6n4p196
198. Pfefferbaum, R., Pfefferbaum, B., Van Horn, R., 2011. Communities AdvancingResilience Toolkit (CART): The CART Integrated System. Terrorism and DisasterCenter at the University of Oklahoma Health Sciences Center, Oklahoma City,OK.
199. Ramsay, T., & Manderson, L. (2011). Resilience, spirituality and posttraumatic growth: reshaping the effects of climate change. In Climate Change and Human Well-Being. New York: Springer. ISBN: 978-1-4419-9742-5
200. Rataj, E., Kunzweiler, K., & Garthus-Niegel, S. (2016). Extreme weather events in developing countries and related injuries and mental health disorders - A systematic review. *BMC Public Health, 16*(1). https://doi.org/10.1186/s12889-016-3692-7
201. Reser, J., Bradley, G., & Ellul, M. (2012). Coping with climate change: Bringing psychological adaptation in from the cold. In B. M. & V. Grimaldo (Ed.), *Handbook of the psychology of coping: Psychology of emotions, motivations and actions* (pp. 1–34). United States: Nova Science Publishers. ISBN: 9781620814642
202. Reser, J. P., Morrissey, S. A., & Ellul, M. (2011a). The threat of climate change: Psychological response, adaptation, and impacts. In Climate Change and Human Well-Being (pp. 19–42). New York: Springer. ISBN: 978-1-4419-9742-5
203. Reser, J. P., & Swim, and J. K. (2011b). Adapting to and coping with the threat and impacts of climate change. *American Psychologist, 66,* 277–289. https://doi.org/doi:10.1037/a0023412
204. Rice, S. M., & McIver, L. J. (2016). Climate change and mental health: Rationale for research and intervention planning. *Asian Journal of Psychiatry, 20*, 1–2. DOI:10.1016/j.ajp.2015.12.011
205. Riemer, M., & Reich, S. M. (2011). Community psychology and global climate change: Introduction to the special section. *American Journal of Community Psychology, 47*(3–4), 349–353. DOI:10.1007/s10464-010-9397-7

206. Rigby, C. W., Rosen, A., Berry, H. L., & Hart, C. R. (2011). If the land's sick, we're sick: The impact of prolonged drought on the social and emotional well-being of Aboriginal communities in rural New South Wales. *Australian Journal of Rural Health, 19*(5), 249–254. https://doi.org/10.1111/j.1440-1584.2011.01223.x
207. Rodriguez-Llanes, J. M., Vos, F., & Guha-Sapir, D. (2013). Measuring psychological resilience to disasters: Are evidence-based indicators an achievable goal? *Environmental Health: A Global Access Science Source, 12*(1). https://doi.org/10.1186/1476-069X-12-115
208. Roeser, S. (2012). Risk communication, public engagement, and climate change: a role for emotions. *Risk Analysis, 32*(6), 1033–1040. https://doi.org/10.1111/j.1539-6924.2012.01812.x
209. Roufeil, L., Gullifer, J., & Maybery, D. (2014). The health challenges facing rural people and communities in Australia: What can psychology offer? *Australian Journal of Rural Health, 22*(6), 271–272. https://doi.org/10.1111/ajr.12155
210. Rudolph, L., & Gould, S. (2015). Climate change and health inequities: A framework for action. *Annals of Global Health, 81*(3), 432–444. https://doi.org/10.1016/j.aogh.2015.06.003
211. Rufat, S., Tate, E., Burton, C. G., & Maroof, A. S. (2015). Social vulnerability to floods: Review of case studies and implications for measurement. *International Journal of Disaster Risk Reduction, 14*, 470–486.https://doi.org/10.1016/j.ijdrr.2015.09.013
212. Sahni, V., Scott, A. N., Beliveau, M., Varughese, M., Dover, D. C., & Talbot, J. (2016). Public health surveillance response following the southern Alberta floods, 2013. *Can J Public Health, 107*(2), 142-148. doi: 10.17269/cjph.107.5188.
213. Saniotis, A., & Irvine, R. (2010). Climate change and the possible health effects on older Australians. *Australian Journal of Primary Health, 16*(3), 217–220. https://doi.org/10.1071/PY10025
214. Sapiains, R., Beeton, R. J. S., & Walker, I. A. (2016). Individual responses to climate change: Framing effects on pro-environmental behaviors. *Journal of Applied Social Psychology, 46*(8), 483–493. https://doi.org/10.1111/jasp.12378
215. Satcher, D., Friel, S., & Bell, R. (2007). Natural and manmade disasters and mental health. *Journal of the American Medical Association, 298*(21), 2540–2542. https://doi.org/10.1001/jama.298.21.2540
216. Schmeltz, M. T., González, S. K., Fuentes, L., Kwan, A., Ortega-Williams, A., & Cowan, L. P. (2013). Lessons from hurricane sandy: A Community response in Brooklyn, New York. *Journal of Urban Health, 90*(5), 799–809. https://doi.org/10.1007/s11524-013-9832-9
217. Schrader, S., & Shattell, M. (2013). Cultural cognition: What mental health researchers and clinicians might learn from the climate change debate. *Issues in Mental Health Nursing, 34*(11), 842–843. https://doi.org/10.3109/01612840.2013.816398
218. Schulte, P. A., Bhattacharya, A., Butler, C. R., Chun, H. K., Jacklitsch, B., Jacobs, T., … Wagner, G. R. (2016). Advancing the framework for considering the effects of climate change on worker safety and health. *Journal of Occupational and Environmental Hygiene, 13*(11), 847–865. https://doi.org/10.1080/15459624.2016.1179388

219. Schulte, P. A., & Chun, H. (2009). Climate change and occupational safety and health: establishing a preliminary framework. *Journal of Occupational and Environmental Hygiene, 6*(9), 542–554. DOI:10.1080/15459620903066008
220. Shultz, J. M., & Galea, S. (2017). Mitigating the mental and physical health consequences of Hurricane Harvey. *Jama*, *318*(15), 1437-1438. doi:10.1001/jama.2017.14618
221. Searle, K., & Gow, K. (2009). Exploring the psychological aspects of risk, fears, and concerns about climate change. *Meltdown: Climate change, natural disasters, and other catastrophes–Fears and concerns of the future*, 31-54.
222. Searle, K., & Gow, K. (2010). Do concerns about climate change lead to distress? *International Journal of Climate Change Strategies and Management, 2*(4), 362–379. https://doi.org/10.1108/17568691011089891
223. Selvey, L. A. (2015). Climate change is harmful to our health: Taking action will have many benefits. *Medical Journal of Australia, 203*(10), 397–398. https://doi.org/10.5694/mja15.00655
224. Shaw, A., Sheppard, S., & Burch, S. (2009). Making local futures tangible-Synthesizing, downscaling, and visualizing climate change scenarios for participatory capacity building. *Global Environmental Change, 19* (4). https://doi.org/10.1016/j.gloenvcha.2009.04.002
225. Shaw, D., Scully, J., & Hart, T. (2014). The paradox of social resilience: How cognitive strategies and coping mechanisms attenuate and accentuate resilience. *Global Environmental Change, 25*(1), 194–203. https://doi.org/10.1016/j.gloenvcha.2014.01.006
226. Sharifi. A. (2016). A critical review of selected tools for assessing community resilience. *Ecological Indicators, 69*, 629–647.https://doi.org/10.1016/j.ecolind.2016.05.023
227. Shukla, J. (2013). Extreme weather events and mental health: Tackling the psychosocial challenge. *ISRN public health, 2013*. http://dx.doi.org/10.1155/2013/127365
228. Silove, D., Steel, Z., & Psychol, M. (2006). Understanding community psychosocial needs after disasters: Implications for mental health services. *Journal of Postgraduate Medicine, 52*(2), 121.
229. Smoyer-Tomic, K. E., Klaver, J. D., Soskolne, C. L., & Spady, D. W. (2004). Health consequences of drought on the Canadian prairies. *EcoHealth, 1*(2), SU144-SU154.
230. Simpson, D. M., Weissbecker, I., & Sephton, S. E. (2011). Extreme weather-related events: Implications for mental health and well-being. In Climate Change and Human Well-Being (pp. 57–78). New York: Springer. ISBN: 978-1-4419-9742-5
231. Spence, A., Poortinga, W., & Pidgeon, N. (2012). The psychological distance of climate change. *Risk Analysis, 32*(6), 957–972. https://doi.org/10.1016/j.envsci.2017.04.011
232. Stain, H. J., Kelly, B., Carr, V. J., Lewin, T. J., Fitzgerald, M., & Fragar, L. (2011). The psychological impact of chronic environmental adversity: Responding to prolonged drought. *Social Science & Medicine, 73*(11), 1593–1599. doi: 10.1016/j.socscimed.2011.09.016
233. Stanke, C., Murray, V., Amlôt, R., Nurse, J., & Williams, R. (2012). The effects of flooding on mental health: Outcomes and recommendations from a review of the literature. *PLoS currents*, *4*.

234. Stedman, R. C. (2004). Risk and climate change: perceptions of key policy actors in Canada. *Risk Analysis, 24*(5), 1395–1406. DOI:10.1111/j.0272-4332.2004.00534.x
235. Stephenson, J., Vaganay, M., Cameron, R., & Joseph, P. (2014). The long-term health impacts of repeated flood events. *WIT Transactions on Ecology and the Environment, 184*, 201–212. https://doi.org/10.2495/FRIAR140171238.
236. Stuart, K., Mahmood, M. A., Clark, L., & Pace, R. (2011). Health education and a co-ordinated response system to support vulnerable people during heat waves. *Australian and New Zealand Journal of Public Health, 35*(4), 394–39 https://doi.org/10.1111/j.1753-6405.2011.00749.x
237. Sung, T.-I., Chen, M.-J., Lin, C.-Y., Lung, S.-C., & Su, H.-J. (2011). Relationship between mean daily ambient temperature range and hospital admissions for schizophrenia: Results from a national cohort of psychiatric inpatients. *Science of the Total Environment, 410*–411, 41–46. https://doi.org/10.1016/j.scitotenv.2011.09.028
238. Swim, J., Clayton, S., Doherty, T., Gifford, R., Howard, G., Reser, J., & Weber, E. (2009). Psychology and global climate change: Addressing a multi-faceted phenomenon and set of challenges. A report by the American Psychological Association's task force on the interface between psychology and global climate change. Washington: American Psychological Association.
239. Swim, J. K., Clayton, S., & Howard, G. S. (2011). Human behavioral contributions to climate change: Psychological and contextual drivers. *American Psychologist, 66*(4), 251. DOI:10.1037/a0023472
240. Swim, J. K., Stern, P. C., Doherty, T. J., Clayton, S., Reser, J. P., Weber, E. U., & Howard, G. S. (2011). Psychology's contributions to understanding and addressing global climate change. *American Psychologist, 66*(4), 241. http://dx.doi.org/10.1037/a0023220
241. Syal, S. S., Wilson, R. S., Crawford, J. M., & Lutz, J. (2011). Climate change and human health-what influences the adoption of adaptation programming in the United States public health system? *Mitigation and Adaptation Strategies for Global Change, 16(*8), 911–924. https://doi.org/10.1007/s11027-011-9302-1
242. Tapsell, S. (2010). Socio-Psychological Dimensions of Flood Risk Management. In Pender, G., & Faulkner, H. (Eds.). (2010). *Flood risk science and management*. John Wiley & Sons. ISBN: 9781444324846
243. 246. Thomas, F., Sabel, C. E., Morton, K., Hiscock, R., & Depledge, M. H. (2014). Extended impacts of climate change on health 'and wellbeing. *Environmental Science and Policy, 44*, 271–278. https://doi.org/10.1016/j.envsci.2014.08.011
244. Thornley, L., Ball, J., Signal, L., Lawson-Te Aho, K., & Rawson, E. (2015). Building community resilience: learning from the Canterbury earthquakes. *Kotuitui, 10*(1), 23–35. https://doi.org/10.1080/1177083X.2014.934846
245. Tierney, K. (2014). The social roots of risk: Producing disasters, promoting resilience. Stanford University Press. 9780804791403.
246. THRIVE, 2004. A Community Approach to Address Health Disparities: Toolkit forHealth & Resilience In Vulnerable Environments. Prevention Institute http://www.preventioninstitute.org/component/jlibrary/article/id-96/127.html.

247. Tobias, J. K., & Richmond, C. A. M. (2014). "That land means everything to us as Anishinaabe. . ..": Environmental dispossession and resilience on the North Shore of Lake Superior. *Health and Place, 29*, 26–33. https://doi.org/10.1016/j.healthplace.2014.05.008
248. Tong, S., Mather, P., Fitzgerald, G., McRae, D., Verrall, K., & Walker, D. (2010). Assessing the vulnerability of eco-environmental health to climate change. *International Journal of Environmental Research and Public Health, 7*(2), 546–564. https://doi.org/10.3390/ijerph7020546
249. Tonna, A., Kelly, B., Crockett, J., Buss, R., Roberts, R., Wright, M., & Greig, J. (2009). Improving the mental health of drought affected communities*. An Australian model. Rural Society, 19*(4), 296–305. https://doi.org/10.5172/rsj.351.19.4.296
250. Tosone, C., McTighe, J. P., & Bauwens, J. (2015). Shared Traumatic Stress among Social Workers in the Aftermath of Hurricane Katrina. *British Journal of Social Work, 45(*4), 1313–1329. https://doi.org/10.1093/bjsw/bct194
251. Trombley, J., Chalupka, S., & Anderko, L. (2017). Climate Change and Mental Health. *AJN The American Journal of Nursing, 117*(4), 44-52. DOI: 10.1097/01.NAJ.0000515232.51795.fa
252. Trang, P. M., Rocklöv, J., Giang, K. B., Kullgren, G., & Nilsson, M. (2016). Heatwaves and hospital admissions for mental disorders in Northern Vietnam e0155609. *PLoS ONE, 11*(5). https://doi.org/10.1371/journal.pone.0155609
253. Truelove, H. B., Carrico, A. R., & Thabrew, L. (2015). A socio-psychological model for analyzing climate change adaptation: A case study of Sri Lankan paddy farmers. *Global Environmental Change, 31*, 85–97. https://doi.org/10.1016/j.gloenvcha.2014.12.010
254. Turner, L. R., Alderman, K., & Tong, S. (2012). The 2011 Brisbane floods affected residents' health. *Medical Journal of Australia, 197*(4), 214–216. https://doi.org/10.5694/mja12.10724
255. Tunstall, S., Tapsell, S., Green, C., Floyd, P., & George, C. (2006). The health effects of flooding: social research results from England and Wales. *Journal of water and health, 4*(3), 365-380.
256. Van Kessel, G., Macdougall, C., & Gibbs, L. (2014). Resilience - Rhetoric to reality: A systematic review of intervention studies after disasters. *Disaster Medicine and Public Health Preparedness, 8*(5), 452–460. https://doi.org/10.1017/dmp.2014.104
257. Vasseur, L., Thornbush, M., & Plante, S. (2015). Gender-based experiences and perceptions after the 2010 winter storms in Atlantic Canada. *International journal of environmental research and public health*, *12*(10), 12518-12529. DOI: 10.3390/ijerph121012518
258. Verplanken, B., & Roy, D. (2013). "My Worries Are Rational, Climate Change Is Not": Habitual Ecological Worrying Is an Adaptive Response. *PLoS ONE, 8*(9). https://doi.org/10.1371/journal.pone.0074708
259. Vida, S., Durocher, M., Ouarda, T. B., & Gosselin, P. (2012). Relationship between ambient temperature and humidity and visits to mental health emergency departments in Quebec. *Psychiatric Services*, *63*(11), 1150-1153. DOI:10.1176/appi.ps.201100485.

260. Vins, H., Bell, J., Saha, S., & Hess, J. J. (2015). The mental health outcomes of drought: A systematic review and causal process diagram. *International Journal of Environmental Research and Public Health, 12*(10), 13251–13275. https://doi.org/10.3390/ijerph121013251
261. Wainwright, T. (2009). Climate change and refocusing clinical psychology. Clinical Psychology Forum, (200), 7–11.
262. Waite, T. D., Chaintarli, K., Beck, C. R., Bone, A., Amlôt, R., Kovats, S., ... & Oliver, I. (2017). The English national cohort study of flooding and health: cross-sectional analysis of mental health outcomes at year one. *BMC public health, 17*(1), 129. https://doi.org/10.1186/s12889-016-4000-2
263. Wang, X., Lavigne, E., Ouellette-kuntz, H., & Chen, B. E. (2014). Acute impacts of extreme temperature exposure on emergency room admissions related to mental and behavior disorders in Toronto, Canada. *Journal of affective disorders*, *155*, 154-161. https://doi.org/10.1016/j.jad.2013.10.042
264. Weissbecker, I. (2011). Climate change and human well-being. Springer Fachmedien. ISBN: 978-1-4419-9742-5
265. Wenden, A. L. (2011). Women and climate change: Vulnerabilities and challenges. In Climate Change and Human Well-Being. New York: Springer. ISBN: 978-1-4419-9742-5
266. West, C., Bernard, B., Mueller, C., Kitt, M., Driscoll, R., & Tak, S. (2008). Mental health outcomes in police personnel after Hurricane Katrina. *Journal of Occupational and Environmental Medicine, 50*(6), 689–695. DOI:10.1097/JOM.0b013e3181638685.
267. White, R. G., Imperiale, M. G., & Perera, E. (2016). The Capabilities Approach: Fostering contexts for enhancing mental health and wellbeing across the globe. *Globalization and Health, 12*(1). https://doi.org/10.1186/s12992-016-0150-3
268. Wilby, R. L., & Keenan, R. (2012). Adapting to flood risk under climate change. *Progress in Physical Geography, 36*(3), 348–378. https://doi.org/10.1177/0309133312438908
269. Wilson, S. M., Richard, R., Joseph, L., & Williams, E. (2010). Climate change, environmental justice, and vulnerability: an exploratory spatial analysis. *Environmental Justice, 3*(1), 13–19. DOI: 10.1089/env.2009.0035
270. Winkler, M. S., Röösli, M., Ragettli, M. S., Cissé, G., Müller, P., Utzinger, J., & Perez, L. (2015). Mitigating and adapting to climate change: a call to public health professionals. International Journal of Public Health, 6, 631-2. DOI:10.1007/s00038-015-0722-7
271. Wolf, J., Allice, I., & Bell, T. (2015). Values and traditional practices in adaptation to climate change. Evidence from a Q method study in two communities in Labrador, Canada. Cambridge University Presshttps://doi.org/10.1017/CBO9781139149389.011
272. Wolkin, A., Patterson, J. R., Harris, S., Soler, E., Burrer, S., McGeehin, M., & Greene, S. (2015). Reducing Public Health Risk during Disasters: Identifying Social Vulnerabilities. *Journal of Homeland Security and Emergency Management, 12*(4), 809–822. https://doi.org/10.1515/jhsem-2014-0104
273. Wulff, K., Donato, D., & Lurie, N. (2015). What is health resilience and how can we build it? *Annual Review of Public Health, 36,* 361–374. https://doi.org/10.1146/annurev-publhealth-031914-122829

274. Yusa, A., Berry, P., J Cheng, J., Ogden, N., Bonsal, B., Stewart, R., & Waldick, R. (2015). Climate change, drought and human health in Canada. *International journal of environmental research and public health*, *12*(7), 8359-8412.DOI:10.3390/ijerph120708359.
275. Zakour, M. J., & Gillespie, D. F. (2013). Community disaster vulnerability. *Theory, Research, and Practice, NY: Springer*.ISBN: 978-1-4614-5737-4
276. Zaremohzzabieh, Z., & Samah, B. A. (2013). A review paper: The role of the internet in promoting youth well-being in flood-prone communities. *Asian Social Science, 9*(11), 75–82. https://doi.org/10.5539/ass.v9n11p75
